# Adamantinomatous craniopharyngioma cyst fluid can trigger inflammatory activation of microglia to damage the hypothalamic neurons by inducing the production of β-amyloid

**DOI:** 10.1186/s12974-022-02470-6

**Published:** 2022-05-07

**Authors:** Yilamujiang Ainiwan, Yiguang Chen, Chaofu Mao, Junxiang Peng, Siyuan Chen, Songtao Wei, Songtao Qi, Jun Pan

**Affiliations:** Department of Neurosurgery, Nanfang Hospital, Southern Medical University, No. 1838, Guangzhou North Road, Guangzhou, Guangdong China

**Keywords:** Adamantinomatous Craniopharyngioma, Cyst Fluid, Hypothalamus, Single-Cell RNA Sequencing, Microglia, Inflammation, β-Amyloid, Growth Retardation, Obesity

## Abstract

**Introduction:**

The mechanism by which adamantinomatous craniopharyngioma (ACP) damages the hypothalamus is still unclear. Cyst fluid rich in lipids and inflammatory factors is a characteristic pathological manifestation of ACP and may play a very important role in hypothalamic injury caused by tumors.

**Objective:**

The objective of this study was to construct a reliable animal model of ACP cyst fluid-induced hypothalamic injury and explore the specific mechanism of hypothalamic injury caused by cyst fluid.

**Methods:**

An animal model was established by injecting human ACP cyst fluid into the bilateral hypothalamus of mice. ScRNA-seq was performed on the mice hypothalamus and on an ACP sample to obtain a complete gene expression profile for analysis. Data verification was performed through pathological means.

**Results:**

ACP cystic fluid caused growth retardation and an increased obesity index in mice, affected the expression of the Npy, Fgfr2, Rnpc3, Sst, and Pcsk1n genes that regulate growth and energy metabolism in hypothalamic neurons, and enhanced the cellular interaction of Agrp–Mc3r. ACP cystic fluid significantly caused inflammatory activation of hypothalamic microglia. The cellular interaction of CD74–APP is significantly strengthened between inflammatory activated microglia and hypothalamic neurons. Beta-amyloid, a marker of neurodegenerative diseases, was deposited in the ACP tumor tissues and in the hypothalamus of mice injected with ACP cyst fluid.

**Conclusion:**

In this study, a novel animal model of ACP cystic fluid-hypothalamic injury was established. For the first time, it was found that ACP cystic fluid can trigger inflammatory activation of microglia to damage the hypothalamus, which may be related to the upregulation of the CD74–APP interaction and deposition of β-amyloid, implying that there may be a similar mechanism between ACP cystic fluid damage to the hypothalamus and neurodegenerative diseases.

**Supplementary Information:**

The online version contains supplementary material available at 10.1186/s12974-022-02470-6.

## Introduction

Adamantinomatous craniopharyngioma (ACP) is a rare epithelial tumor that originates from the residual cells of the Rathke sac during embryonic development [1, 2]. Its occurrence is associated with activation of the Wnt/β-catenin signal transduction pathway, which promotes the occurrence and development of tumors [2]. ACP is mainly seen in children, and the cystic formation is a typical pathological characteristic which occurs on over 90% of ACP patients [2–4]. ACP is a benign tumor of WHO grade I. At present, total resection is still the main treatment method. However, follow-up data from a large number of ACP cases show that there is a widespread severe quality of life impairment among the survivors , including growth retardation and obesity, which are mainly related to hypothalamic damage [3, 4].

The hypothalamus, which regulates energy metabolism [5, 6] and body growth [7, 8], is vulnerable to injury due to its close proximity to the ACP [9]. The arcuate nucleus (ARC), an important hypothalamic nucleus that mainly includes neuroendocrine cells and concentrated projection neurons [6, 10, 11], could regulate GHRH neurons (growth hormone-releasing hormone, GHRH) and SST neurons (somatostatin) to control the secretion mode of growth hormone [12]. At the same time, ARC activates or inhibits neuropeptide Y (NPY) and agouti-related protein (AgRP) to control feeding behavior and energy metabolism [13], which is closely related to the onset of obesity and type 2 diabetes [14].

ACP is mostly composed of cyst fluid and its surrounding cyst wall, with a few substantial components [15]. The composition of cyst fluid is complex and usually rich in cholesterol and lipids, as well as various proinflammatory factors [16–18]. Our team discovered in previous studies that the production of ACP cystic fluid may be related to the disorder of lipid metabolism in tumor cells [15]. Lipid metabolism disorder was closely related to inflammation of the hypothalamus [19, 20]. Compared with those in other brain tumors, the levels of inflammatory factors in ACP cystic fluid and solid components are increased [21]. It has been reported that the leakage of cyst fluid can cause severe chemical meningitis [22], and it was previously reported that the injection of cyst fluid into the cortex of rats caused increased expression of markers of inflammation and cell damage and increased body weight [23]. In vitro experiments have shown that cyst fluid can cause degenerative changes and apoptosis of neurons [24]. More and more data proved that ACP cystic fluid may be a key factor of hypothalamic inflammatory injury. However, there is currently no reliable ACP cystic fluid-hypothalamus injury animal model for in-depth study of the specific mechanism by which cyst fluid induces hypothalamic injury. The effect of cystic fluid, which is the product of ACP lipid metabolism disorder, on the hypothalamus is still unclear.

The lack of stable cell lines and benign characteristics make it difficult for ACP cells to form the transplanted tumor in hypothalamus; therefore , a study on the cystic fluid, product of ACP cells lipid metabolism disorder, is very necessary and feasible. To explore the effect of ACP cystic fluid on the hypothalamus, this study used stereotactic technology to inject human ACP cystic fluid into the bilateral hypothalamus of 6-week-old male C57 mice as the cystic fluid group, injected the same amount of PBS as the sham operation group, a blank control group received no treatment. After 8 weeks, ACP cystic fluid caused growth retardation, increased the obesity index, and decreased plasma GH and GHRH levels. To determine whether changes in the expression of genes regulating body growth and energy metabolism occurred in hypothalamic neurons, we performed single-cell RNA sequencing on the hypothalamus of mice from the cystic fluid group and the sham operation group and found that ACP cyst fluid significantly affected the expression of the Sst, Fgfr2, and Rnpc3 genes, which regulate body growth and development [12, 25–28], and the expression of the Npy and Pcsk1n genes, which regulate energy metabolism in hypothalamic neurons [13, 14, 29–33], and caused a significant upregulation of the Agrp–Mc3r cellular interaction, which regulate food intake [34–36], between Agrp/Npy neurons and Ghrh neurons. We unexpectedly found that ACP cystic fluid caused inflammatory activation of hypothalamic microglia. We found for the first time that the cellular interaction of CD74–APP, previously found in neurodegenerative diseases [37], is significantly strengthened between inflammation activated microglia and hypothalamic neurons. Using single-cell RNA sequencing, we also found the same phenomenon of inflammatory activation of microglia in ACP tumor tissue as in mice. Aβ, a marker of neurodegenerative diseases [37, 38], is deposited in the hypothalamus of mice injected with ACP cyst fluid and in ACP tumor tissues (Additional files 1, 2, 3, 4).

We report here that ACP cystic fluid induces inflammatory activation of mouse hypothalamic microglia. Activated microglia may mediate a mechanism similar to that observed in neurodegeneration of hypothalamic neurons and participate in this process, which may damage the hypothalamus, leading to growth retardation and obesity.

## Materials and methods

Details are provided in Additional file 5.

1. Animals: Young 6-week-old C57 male mice weighing 19–20 g were screened as experimental subjects. After stereotactic surgery, the mice were placed in the same environment as before the operation and fed normal food and water. The average food intake, average water intake, and average weight of each group of the mice were recorded every 3 days, and the body length (distance from the tip of the nose to the anus) was recorded every 7 days. The above indicators were recorded to 8 weeks after the operation, and the rodent obesity formula was used to calculate the obesity index [39] (Lee index: body weight^1/3^ divided by body length). All animals were performed in accordance with The Basel Declaration. All operations were approved by the Laboratory Animal Center and the Ethics Committee of Southern Medical University and complied with the National Institute of Health Guidelines for the Protection and Use of Laboratory Animals.
2. Collection of childhood ACP cystic fluid and tumor tissue: Childhood ACP (5–12 years old) patients, whose hypothalamus were infringed by the tumor, were screened and the ACP cystic fluid and the ACP tissue with gliosis zone were collected from clinical surgery. These children received tumor resection and surgery at the Neurosurgery Department of Southern Hospital of Southern Medical University. The ACP cystic fluid was collected with a syringe during the surgery, and the cerebrospinal fluid and blood were not mixed. Tumor tissues and cystic fluids were performed in accordance with the Declaration of Helsinki. The patients’ parents all signed an informed consent form expressing their willingness to allow the use of the excised tumor tissue and cystic fluid for scientific research, and the study received approval from the Ethics Committee of Southern Medical University. The collected tumor tissue and cystic fluid from surgery were immediately stored in a freezer at -80 °C. Finally, we collected tumor tissues and cystic fluid from 8 pediatric patients.
3. Stereotactic surgery: The mice were anesthetized by inhalation with isoflurane (2%, 2 L O2/min), and the head was fixed with a stereotaxic instrument (Reward, Shanghai, China). The skull was milled out in the target area, the microsyringe was filled with 20 µl of cystic fluid or PBS, and 10 µl was injected into the hypothalamus on each side. Cystic fluid group: ACP cystic fluid was injected into the bilateral hypothalamus. Sham operation group: Same amount of PBS was used to inject into the bilateral hypothalamus. Control group: No treatment was administered. Each group included 8 mice, and each mouse in the cystic fluid group was injected with cystic fluid from a different patient.
4. Single-cell RNA sequencing: Eight weeks after the operation, a mouse was randomly selected from the cystic fluid group and the sham operation group, respectively. After anesthetization, their hypothalamus was removed immediately under a light microscope for single-cell RNA sequencing. At the same time, a sample of fresh childhood ACP tumor tissue (with gliosis zone) collected during clinical surgery was also used for single-cell RNA sequencing. Details are provided in Additional file 6.
5. Immunohistochemistry and immunofluorescence: Eight weeks after surgery, the mice were anesthetized and perfused transcardially with saline, followed by internal fixation with 4% paraformaldehyde in PBS (phosphate-buffered saline, pH = 7). The brain was removed and soaked with 4% formaldehyde for 48 h. The entire hypothalamus was coronally cut out with stainless steel brain matrices (BB-NMJ), and then 2 µm sections were made. After dewaxed with xylene and alcohol (100%–70%), washed with PBS (pH = 7), and EDTA (pH = 9) for antigen retrieval, the sections were incubated with primary antibody at 4 °C overnight. And then incubated with secondary antibody for 1 h at room temperature. The sections were observed and images were captured under an Olympus microscope (Olympus BX63, Japan).
6. ELISA test for plasma hormone levels: Eight weeks after the operation, the mice were anesthetized with isoflurane, and then 600 µl of blood was drawn from the tail veins to collect serum. Serum was used immediately for ELISA. Samples were diluted and subjected to standard ELISA to determine GH and GHRH levels in accordance with the manufacturer's protocol (mouse GH ELISA kit, EZRMGH-45 K; mouse GHRH ELISA kit, ELK2562; Millipore, Billerica, MA, USA).
7. Mouse microglia (BV2) were stimulated with cystic fluid in vitro. In vitro, cystic fluid was added to culture medium at a ratio of 1:20, in which mouse microglia (BV2) were cultured for 24 h at 37℃.The control group of BV2 cells was cultured in saline-mixed culture medium (saline:culture medium = 1:20) for 24 h at 37℃.
8. RT-qPCR test: Total RNA was extracted from the mouse hypothalamus. The quantity and concentration of RNA were assessed by measuring absorbance. The reverse transcription reactions were performed. Then RT-qPCR was performed following the manufacturer's instructions. The relative expression levels of each sample were calculated using the 2^−ΔΔCt^ method with glyceraldehyde-3-phosphate dehydrogenase (GAPDH) as the endogenous control.
9. Statistical analysis. All values are expressed as the mean ± SEM and represent data from at least 3 repeated experiments. Data were analyzed using SPSS 22 (IBM, MIT) and GraphPad (San Diego, CA) software. If the mean satisfied the normal distribution, a parameter test was used, one-way ANOVA was used for data with a homogeneous variance, and Welch ANOVA was used for data with a heterogeneous variance. If the normal distribution was not satisfied, the Kruskal–Wallis test was used. P < 0.05 (*), P < 0.01 (**), P < 0.001 (***), and P < 0.0001 (****) were considered to indicate statistical significance. NS (P > 0.05) means that there was no significant difference.

## Results

### ACP cystic fluid caused growth retardation and increased the obesity index in mice

**Fig. 1:**
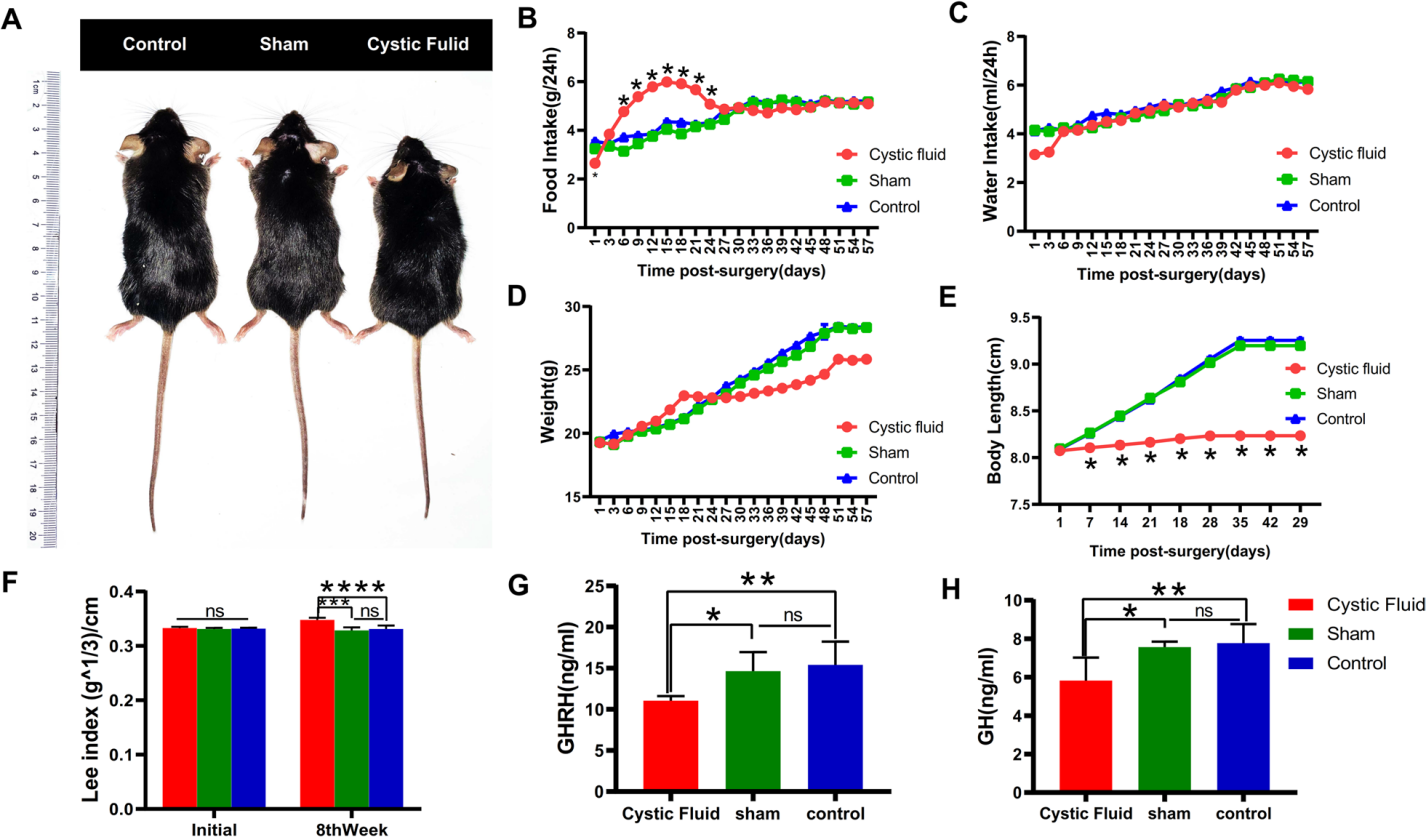
ACP cystic fluid caused growth retardation and increased the obesity index in mice. **A** Eight weeks after surgery, the phenotypes of the 3 groups of mice were assessed. **B** A graph of average food intake after surgery. Mice in the cystic fluid group showed increased food intake in the early stage after surgery. **C** A graph of the average water intake after surgery. There was no difference in the amount of water consumed by the three groups of mice. **D** A graph showing the body weight change after the operation. The weight gain of mice in the cystic fluid group was faster at the initial stage after surgery and then became slower. After 8 weeks, their body weight was smaller than that of the sham-operated group and the control group. **E** Curve chart of body length change after surgery. The average body length of mice in the cystic fluid group hardly increased within 8 weeks after surgery. **F** There was no difference in the initial obesity index (Lee index) of the three groups of mice. The obesity index of the mice in the cystic fluid group was higher than that of the sham operation group and the control group at 8 weeks after surgery (*P* < 0.001). **G-H**. Eight weeks after surgery, the plasma GHRH and GH levels of mice in the cystic fluid group were lower than those in the sham operation group and the control group (*P* < 0.05). Control: *N* = 8, sham: *N* = 8, cystic fluid: *N* = 8

The body weight, food intake, water intake, and body length of each group of mice were dynamically recorded from the first day after the injection of ACP cystic fluid. After 8 weeks, we found that the cystic fluid group had a short stature phenotype (Fig. 1A). The body length of mice in the cystic fluid group was significantly smaller than that of the sham operation group and the control group (*P* < 0.05) (Fig. 1E). Although the body weight of the mice in the cystic fluid group was also smaller than that of the sham operation group and the control group, there is no significant difference (*P* > 0.05, Fig. 1D). The obesity index (Lee index) of the cystic fluid group was higher than that of the sham operation group and the control group (*P* < 0.05, Fig. 1F). We found that the food intake of the cystic fluid group was greater than that of the sham operation group and the control group on the 6th day after surgery (*P* < 0.05); this trend continued until the 24th day after the surgery, and the food intake then gradually decreased to a level similar to that of the sham operation group and the control group and was stable until 8 weeks after the operation (Fig. 1B). 6 to 24 days after the operation, the cystic fluid group also experienced a period of rapid weight gain, and there was almost no weight gain afterward (Fig. 1D). Surprisingly, the body length of the cystic fluid group hardly increased within 8 weeks after surgery (Fig. 1E). There was no difference in water consumption among the three groups within 8 weeks (*p*>0.05, Fig. 1C). There was no significant difference in weight, body length, or obesity index between the control group and the sham operation group (p>0.05). GHRH, secreted by the hypothalamus acts on the pituitary gland, promotes pituitary synthesis and releases GH; GH promotes bone growth and visceral development [40], and GH deficiency can cause severe growth retardation [41]. As a result, we found that the plasma GH and GHRH levels of mice in the cystic fluid group decreased significantly (*P* < 0.05, Fig. 1G, H).

### Single-cell RNA sequencing showed that ACP cystic fluid significantly affects the expression of genes that regulate energy metabolism and body growth in the mouse hypothalamus

**Fig. 2:**
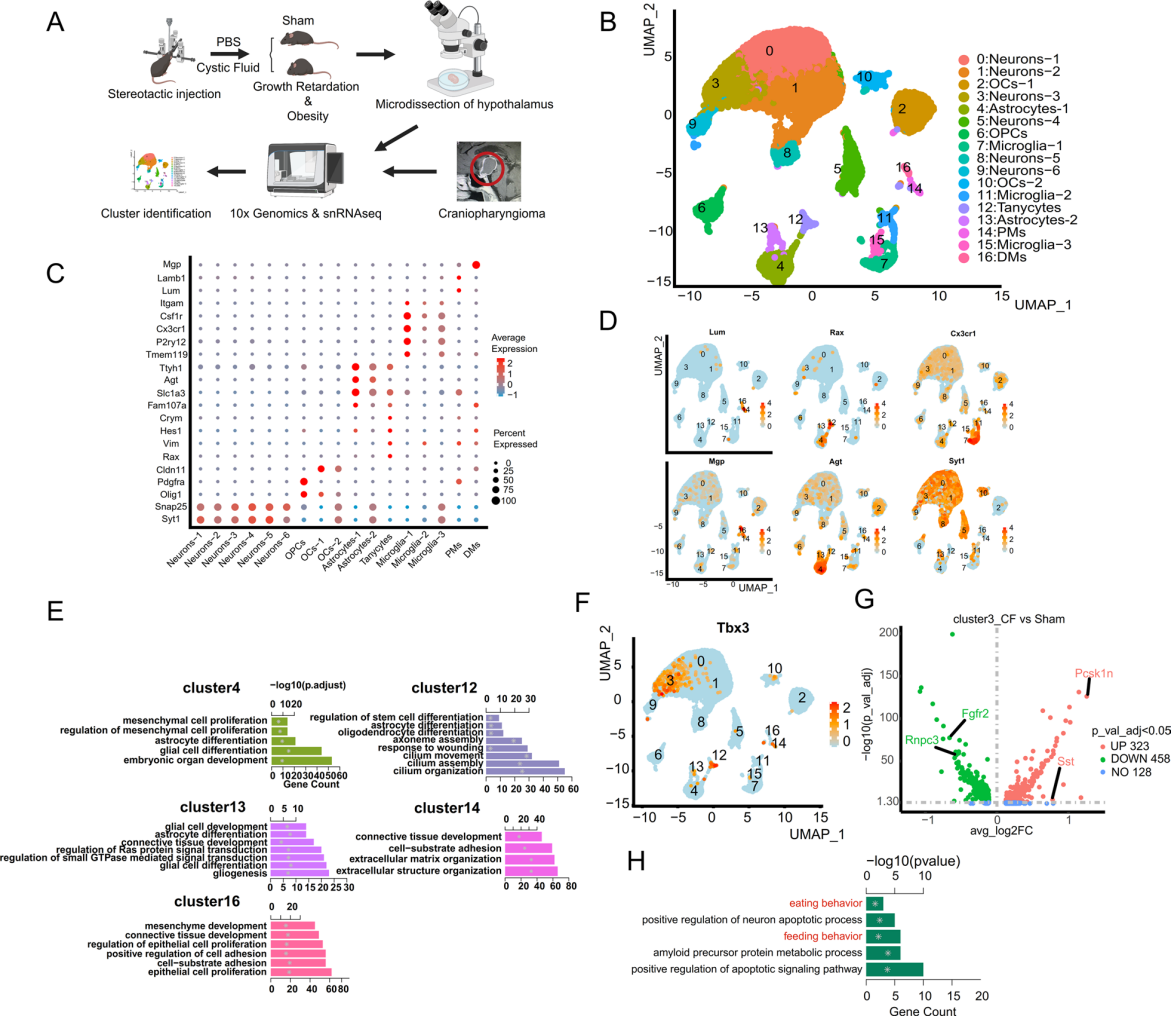
Single-cell RNA sequencing on mouse hypothalamus after injected with ACP cystic fluid or PBS. **A** Schematic diagram of the establishment of the ACP cystic fluid-hypothalamus injury animal model and single-cell RNA sequencing of mouse hypothalamus and childhood ACP tissues. **B** UMAP image of single-cell RNA sequencing of the mouse hypothalamus. According to known specific marker genes, neurons (clusters 0, 1, 3, 5, 8, and 9), oligodendrocytes (clusters 2 and 10), oligodendrocyte premise cells (cluster 6), astrocytes (clusters 4 and 13), tanycytes (cluster 12), microglia (clusters 7, 11, and 15), pia mater cells (cluster 14), and dural cells (cluster 16) were identified. **C** Marker genes specifically expressed in each cell population and the corresponding cell population. Neurons (snap25+/syt1+), microglia (csf1r+/cx3cr1+), astrocytes (agt+), oligodendrocytes (cldn11+), oligodendrocyte precursor cells (pdgfra+/olig1+), tanycytes (rax+/crym+), dural cells (mgp+), and pia mater cells (lum+). **D** A portion of the feature plot analysis results, which allows intuitive visualization of the expression of specific marker genes in specific clusters. **E** GO enrichment map of some clusters. Further clarification of the identity of astrocytes (clusters 4 and 13), tanycytes (cluster 12), pia mater cells (cluster 14), and dural cells (cluster 16). **F** The ARC-specific marker gene TBX3 is highly expressed in cluster 3. **G** In cluster 3, CF (cystic fluid) vs. sham, the expression of the Pcsk1n and Sst genes in the cystic fluid group was significantly upregulated, and the expression of the Fgfr2 and Rnpc3 genes was significantly downregulated. **H** The number of genes related to feeding behavior, positive regulation of neuronal apoptosis, and APP metabolic regulation in cluster 3 of the cystic fluid group was significantly higher than that of cluster 3 of the sham operation group

To further clarify the causes of the growth retardation and the increased obesity index observed in the cystic fluid group, we performed single-cell RNA sequencing on the hypothalamus of the mice from the cystic fluid group and the sham operation group (Fig. 2A). After filtering low-quality samples and cells, 23073 cells were obtained. The median UMI of cystic fluid group was 4153/cell, and the median number of genes per cell was 2168.5/cell. The median UMI of sham operation group was 4749/cell, and the median number of genes per cell was 2477/cell. As a result, 24808 genes were detected in all cells. After removing all the mitochondrial genes through an unsupervised strategy, clustering was performed, and 16 populations were found (Fig. 2B). Through the expression of known markers expressed by some specific hypothalamic cell clusters [42–46], we determined the cell type represented by each cluster; that is, clusters 0, 1, 3, 5, 8, and 9 were neurons (snap25+/syt1+), clusters 7, 11, and 15 were microglia (csf1r+/cx3cr1+), clusters 4 and 13 were astrocytes (agt+), clusters 2 and 10 were oligodendrocyte glial cells (cldn11+), cluster 6 was oligodendrocyte precursor cells (pdgfra+/olig1+), cluster 12 was tanycytes (rax+/crym+), cluster 16 was dural cells (mgp+), and cluster 14 was pia mater cells (lum+) (Fig. 2C). We partially show the results of our in-depth analysis, and we can intuitively see the specific expression of these markers in the corresponding cell population (Fig. 2D). Moreover, in the GO enrichment of cluster 4 and cluster 13, the number of genes related to the differentiation of astrocytes was very high, indicating that these cells represent astrocytes [42]. In the GO enrichment of cluster 12, the number of genes regulating the differentiation of stem cells was very high, which is unique to hypothalamic tanycytes [42]. The GO terms enriched in cluster 14 and cluster 16 were found to be related to the composition of the extracellular matrix and cell connections and supporting tissues, which suggests that these cells may be meningeal cells [42, 44] (Fig. 2E). The number of cells in clusters 14 and 16 of the cyst fluid group was significantly higher than that of the sham operation group, which may be caused by the mixing of meningeal tissue during the removal of the hypothalamus, but this did not affect the experimental results. Ultimately, the identification of all cell populations was completed.

In the hypothalamus, neurons that control body growth [5–8] and energy metabolism [13, 14] are mainly located in the ARC nucleus. TBX3, a marker gene specifically expressed in the ARC [47, 48], was also specifically and highly expressed in cluster 3 (Fig. 2F). Therefore, the cluster 3 comes from the ARC nucleus. As a result, we found that there were significant changes in some genes that regulate body growth and energy metabolism in cluster 3 from the cystic fluid group, and these genes were Fgfr2, Rnpc3, Sst, and Pcsk1n (Fig. 2G). In the cystic fluid group, the expression of the Fgfr2 gene was significantly downregulated in cluster 3. Previous studies have shown that mice exhibit significant growth retardation after the Fgfr2 gene is knocked out [25]. Mutation of the Fgfr2 gene is related to human dwarfism, and this gene was highly conserved during the evolution of different species [25, 26]. In the cystic fluid group, the expression of the RNPC3 gene in cluster 3 was also significantly downregulated. Studies have confirmed that mutations in the Rnpc3 gene cause severe isolated growth hormone deficiency in humans [27, 28]. Additionally, in the cystic fluid group, the expression of the Sst gene was significantly upregulated in cluster 3. The growth inhibitory hormone encoded by the SSt gene is an antagonist of growth hormone and inhibits growth [12]. Studies have shown that the activation of SSt neurons in the hypothalamus promotes food intake and obesity in mice [49]. In the cystic fluid group, the expression of the Pcsk1n gene in cluster 3 was also significantly upregulated. As a natural endogenous inhibitor of Pcsk1 (prohormone converting enzyme 1), Pcsk1n slows down the mediating effect of hormone converting enzyme and promotes food intake and obesity [29, 33], and Pcsk1 gene mutations are common in the obese human population [30–32]. At the same time, the number of GO-enriched genes related to eating behavior, feeding behavior, and apoptosis of cluster 3 in the cystic fluid group was significantly higher than that of cluster 3 in the sham operation group (Fig. 2H), further suggesting that ACP cystic fluid significantly affects the expression of genes that regulate energy metabolism. Subsequently, in PCR analysis of the mouse hypothalamus, we also found that the expression of Fgfr2 and Rnpc3 in the cystic fluid group was significantly decreased, and the expression of Sst and Pcsk1n was significantly increased (Additional file 1: Fig. S1D). The changes in the expression of the above genes may be the cause of growth retardation and increased obesity index in these mice.

### ACP cystic fluid significantly affects the cellular interaction between neurons in the mouse hypothalamus

**Fig. 3:**
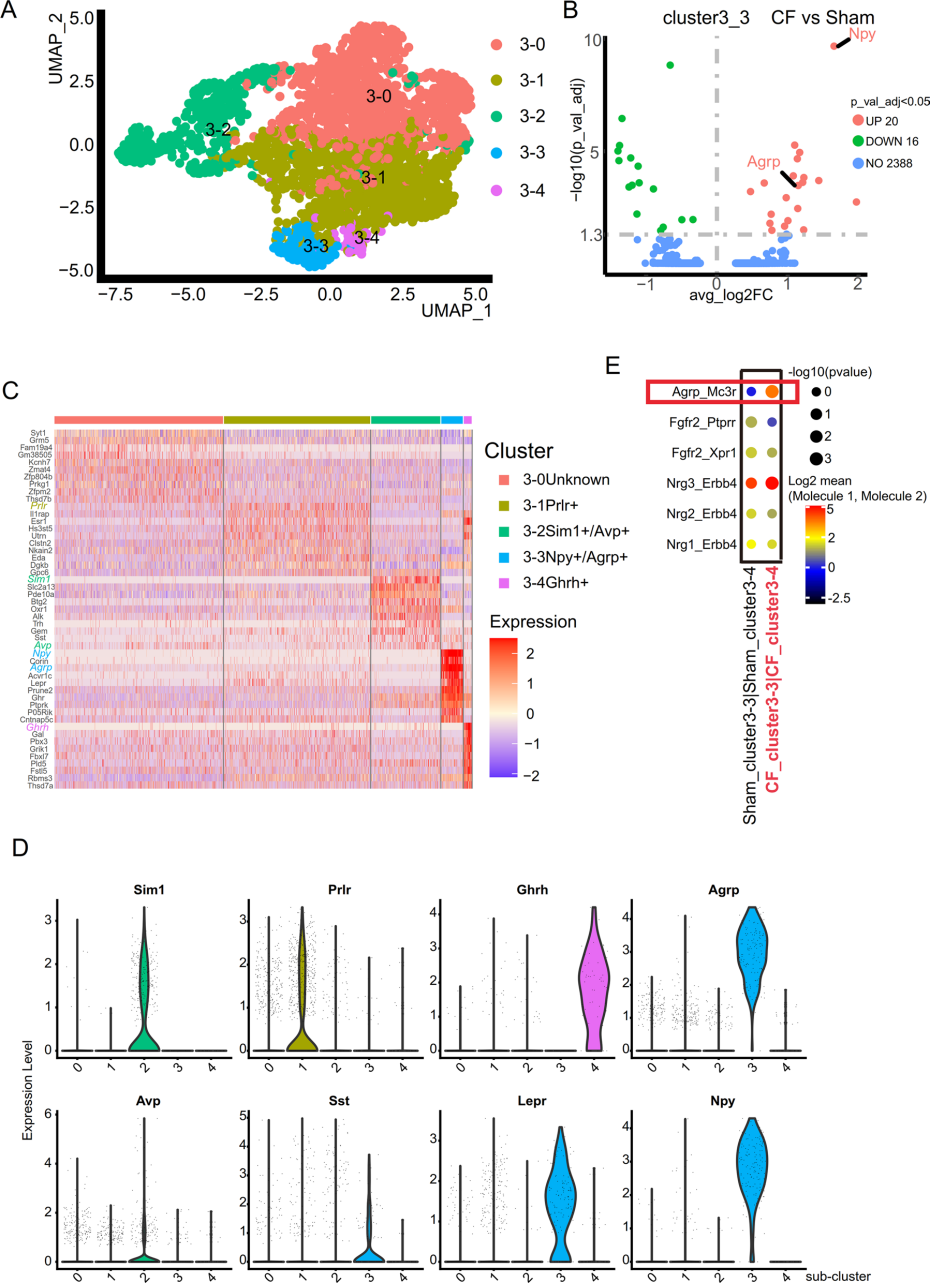
ACP cystic fluid significantly affected the cell interaction between neurons in the mouse hypothalamus. **A** UMAP diagram for classification of the subclusters of cluster 3. **B** The expression of the Agrp gene and Npy gene in subcluster 3–3 (Agrp/Npy neurons) of the cystic fluid group was significantly higher than that of subcluster 3–3 (Agrp/Npy neurons) of the sham operation group. **C** Heatmap of the specific expression of ARC-specific marker genes in each subcluster. The 3–0 subcluster has no specific marker. The Prlr gene is specifically expressed in the 3–1 subcluster, the Sim1 gene and the Avp gene are specifically expressed in the 3–2 subcluster, the Agrp gene and the Npy gene are specifically expressed in the 3–3 subcluster, and the Ghrh gene is specifically expressed in the 3–4 subcluster. **D** Violin diagram of the marker genes specifically expressed in each subcluster of cluster 3. The Sst gene is specifically expressed in the 3–3 subcluster (Agrp/Npy neurons). **E** Intercellular interaction of Agrp–Mc3r is significantly enhanced between subcluster 3–3 (Agrp/Npy neurons) and subcluster 3–4 (Ghrh neurons) in the cystic fluid group (CF). In the interaction, Agrp/Npy neurons express the ligand gene Agrp, and Ghrh neurons express the receptor gene Mc3r

We discovered for the first time that ACP cystic fluid significantly changed the expression of genes regulating body growth and energy metabolism in neurons (cluster 3) from the ARC. Based on this finding, we conducted further studies on cluster 3. We found that in addition to the specific expression of Tbx3 (marker of ARC), cluster 3 also specifically expressed Prlr, Npy, Agrp, and Ghrh, which are also ARC-specific marker genes [42]. Therefore, we classified cluster 3 based on these known marker genes, and 5 subclusters were separated (Fig. 3A). They are subcluster 3-0 (no specific markers but not named), subcluster 3-1 (Prlr neurons (Prlr+)), subcluster 3-2 (Sim1/Avp neurons (Sim1+, Avp+)), subcluster 3-3 (Agrp/Npy neurons (Agrp+, Npy+)), and subcluster 3-4 (Ghrh neurons (Ghrh+) (Fig. 3C). We found that the Sst gene was specifically expressed in Agrp/Npy neurons (Fig. 3D), while POMC and TBX3 exhibited scattered expression in various subclusters of cluster 3 (Additional file 1: Fig. S1F). After subcluster classification was completed, we were surprised to find that in Agrp/Npy neurons, the expression of the Agrp and Npy genes in the cystic fluid group was significantly higher than that in the sham operation group (Fig. 3B). We verified the high expression of Npy, which has the most obvious expression difference, in the mouse hypothalamus by immunohistochemistry (Additional file 1: Fig. S1C). To further clarify the reason for the upregulation of Npy, we conducted a study on the interaction relationship between different subclusters and found that the cell interaction between Agrp/Npy neurons (3-3 subclusters) and Ghrh neurons (3-4 subclusters) in the cystic fluid group was significantly strengthened compared with that in the sham operation group (Fig. 3E). In the cell interaction, Agrp is the ligand gene, Agrp/Npy neurons (subcluster 3-3s) are the cells expressing the ligand gene, Mc3r is the receptor gene, and Ghrh neurons (subcluster 3-4) are the cells expressing the receptor gene. Agrp is an endogenous antagonist of Mc3r [34–36]. Agrp combines with Mc3r to effectively promote feeding behavior and obesity [36]. This result suggests that the increased obesity index and the high expression of Npy in the hypothalamus may be related to the upregulation of the Agrp–Mc3r interaction.

### Single-cell RNA sequencing indicated that mouse hypothalamic microglia underwent inflammatory activation in response to ACP cystic fluid

**Fig. 4:**
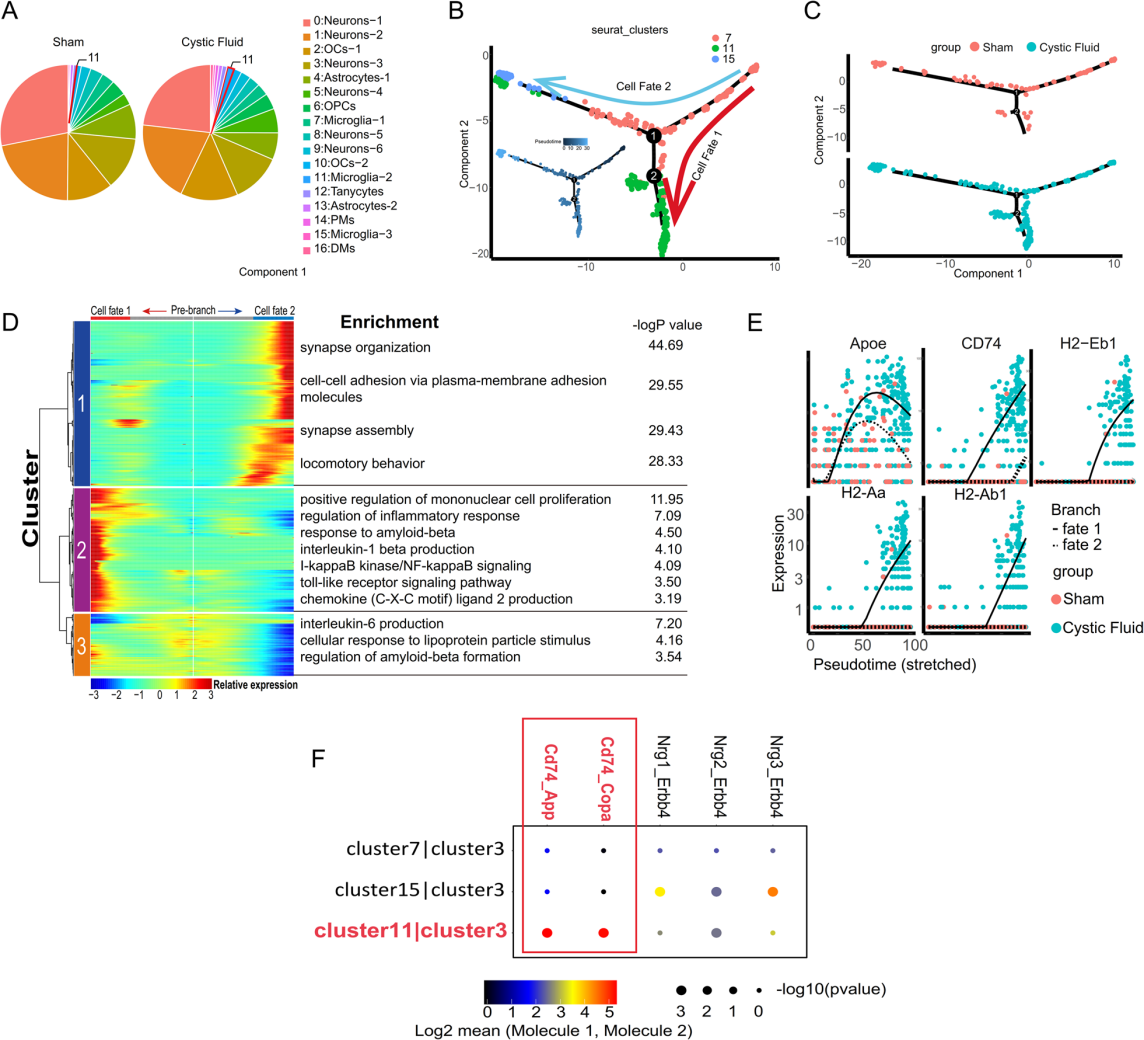
Mouse microglia underwent inflammatory activation in response to ACP cystic fluid and acted on neurons. **A** The number of cluster 11 microglia in the cystic fluid group was far greater than that in the sham operation group. **B** Pseudotime diagram of microglia. A darker color represents the default initial cell point. Microglia gradually differentiated in two directions from the cluster 7. The red line represents the trajectory corresponding to the development of cell fate 1, and the blue line represents the trajectory corresponding to the development of cell fate 2. The developmental trajectory of cell fate 1 is mainly contributed by cluster 11. **C** Branch point 1 to the left corresponds to cell fate 2, and branch point 1 to the downward corresponds to cell fate 1. The developmental trajectory of cell fate 1 is mainly contributed by the microglia of the cyst fluid group. **D** Monocle2's BEAM function was used to analyze the difference of the cells in the two fate stages before and after differentiation at branch point 1, and a heatmap of the differentially enriched genes was drawn to obtain 3 clusters. The GO enrichment analysis showed that the microglia in the developmental trajectory of cell fate 1 mainly exhibit inflammatory activation, increased synthesis of proinflammatory factors, activation of inflammation-related pathways, stimulation of lipoprotein particles, and increased synthesis and response of beta-amyloid (Aβ). **E** The expression of APOE, CD74, H2-Eb1, H2-Aa, and H2-Ab1 genes in the microglia of the developmental trajectory of cell fate1 was upregulated, and the upregulation of these genes was mostly contributed by the cyst fluid group. **F** The intercellular interaction of CD74–App and CD74–COPA was significantly strengthened between neurons of the ARC (cluster 3) and microglia (cluster 11) from the developmental trajectory of cell fate 1. In the interaction relationship, cluster 11 expressed the ligand gene CD74, and cluster 3 expressed the receptor genes APP and COPA

It was previously reported that ACP cyst fluid may have a direct toxic effect on neurons [24], and we also believe that various types of glial cells in the hypothalamic tissue have an effect on neurons. Through further exploration of the single-cell RNA sequencing data, we unexpectedly found that the cell number of cluster 11 microglia in the cystic fluid group was far greater than that in the sham operation group. The cell numbers of cluster 7 microglia and cluster 15 microglia, astrocytes, oligodendrocytes, and oligodendrocyte precursor cells did not show significant differences between the two groups (Fig. 4A, Additional file 1: Fig. S1E). At the same time, we found that cluster 7 microglia and cluster 15 microglia expressed the marker genes of mature microglial cells, such as P2ry12, Cx3cr1, and Tmem119 (Fig. 2C) [50], but the abovementioned mature microglial marker genes showed relatively low expression in cluster 11 microglia (Microglia-2), so we inferred that cluster 11 microglia may be formed by the differentiation of ordinary mature microglia under pathological conditions [51]. To further verify the origin of the cluster 11 microglia, we conducted a pseudochronological analysis of all the microglial clusters identified in the single-cell data. Pseudotime is a pseudochronological diagram of microglial cells. The larger the pseudotime value is, the lighter the color, which represents a greater distance from the initial cell point, and a darker color represents the default initial cell point (Fig. 4B). The starting point here was calculated with Monocle software. Microglia can gradually differentiate in two directions from cluster 7. The red line is the trajectory corresponding to the development of cell fate 1, and the blue line is the trajectory corresponding to the development of cell fate 2 (Fig. 4B). The developmental trajectory of fate 1 is mainly contributed by cluster 11 microglia cells, while fate 2 is the common contribution of cluster 11 and cluster 15 cells (Fig. 4B). At the same time, fate 1 is mainly contributed by the microglia of the cystic fluid group (Fig. 4C). We used monocle2's BEAM function to perform differential analysis of the cells of the two fates before and after differentiation at branch point 1, drew a heatmap of the differentially enriched genes to obtain 3 clusters, and performed individual GO enrichment analysis. GO enrichment analysis of the enriched clusters 2 and 3 revealed that the microglia of the cell fate 1 developmental track mainly show inflammation activation, increased synthesis of proinflammatory factors, activation of inflammation-related pathways, increased response to lipoprotein granule stimulation, and increased beta-amyloid (Aβ) synthesis(Fig. 4D). It is worth noting that beta-amyloid (Aβ) is a pathological sign of Alzheimer’s disease [37, 38]. Subsequently, we verified the high expression of CD68, a marker of microglial activation, and IL6 in the hypothalamus of mice from the cystic fluid group (Additional file 2: Fig. S2C-D). In vitro experiments also verified that cyst fluid promotes the activation of microglia (Additional file 2: Fig. S2A). Interestingly, we also found that in addition to the upregulated expression of genes related to microglial inflammatory activation (H-2 gene), genes related to neurodegenerative diseases, such as CD74 and APOE [52, 53], were also upregulated in the hypothalamus of mice in the cystic fluid group (Fig. 4E).

### Microglia activated by ACP cystic fluid may cause inflammatory damage to hypothalamic neurons

The GO enrichment of cluster 3 and its subclusters in the cystic fluid group indicated that the activation of pathways related to neuronal apoptosis and APP (precursor of Aβ) processing was significantly higher than that in the sham operation group (Fig. 2H, Additional file 1: Fig. S1B). Therefore, to clarify whether the inflammatory activation of microglia is related to neurons, we conducted a study on the cell interaction between microglia and cluster 3 neurons. As a result, we found that the cell–cell interactions mediated by CD74–APP and CD74–COPA were significantly enhanced in the communication between cluster 11 microglia, which have undergone inflammatory activation, and cluster 3 neurons (Fig. 4F). In the interaction relationship, cluster 11 microglia express the ligand gene CD74, and cluster 3 neurons express the receptor genes APP and COPA. Previous reports suggested that the interaction between APP, COPA, and CD74 is often associated with neurodegenerative diseases, such as Alzheimer's disease [37, 38, 52, 54]. We also verified the deposition of β-amyloid (Aβ) in the ARC of mice from the cyst fluid group (Fig. 6A). These results suggested that the mechanism by which microglia that have undergone inflammatory activation in response to ACP cystic fluid act on neurons may be similar to the mechanisms associated with neurodegenerative diseases, inducing neuronal apoptosis.

### The same inflammatory activation of microglia observed in the mouse hypothalamus also occurred in childhood ACP tissue

**Fig. 5:**
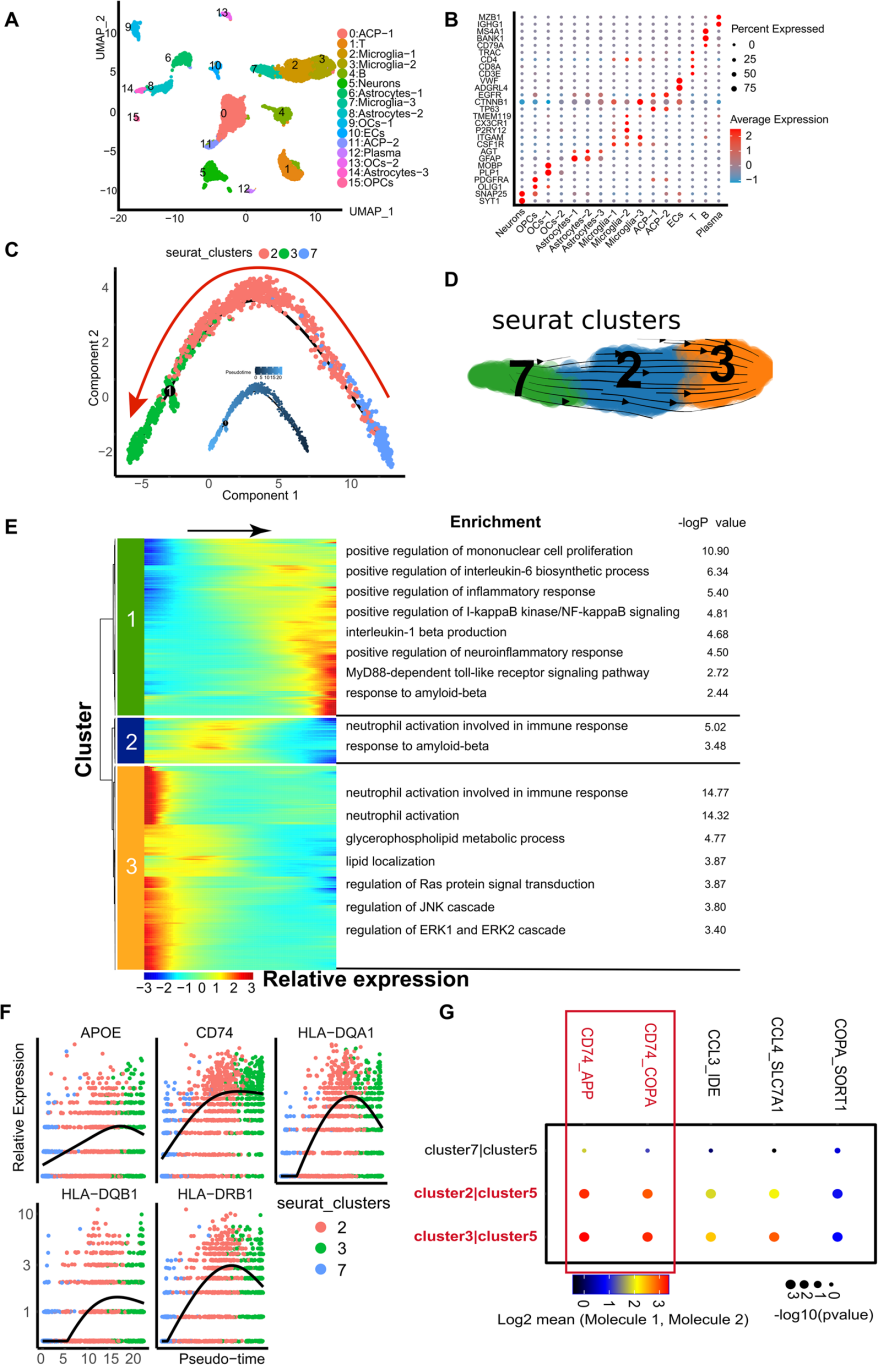
Single-cell RNA sequencing on the childhood ACP tissue. **A** UMAP image of single-cell RNA sequencing of childhood ACP tissue (with gliosis zone). According to the known specific marker genes, 16 cell clusters were identified. **B** The following marker genes were specifically expressed in each corresponding cell cluster: ACP cells (TP63+), neurons (snap25+/syt1+), microglia (csf1r+/cx3cr1+), astrocytes (agt+/gfap+), oligodendrocytes (Plp1+), pre-oligodendrocytes (pdgfra+/olig1+), T lymphocytes (Trac+/CD8A+/CD3E+), B lymphocytes (Bank1+/Ms4a1+/CD79a+), endothelial cells (Vwf+/Adgrl4+), and plasma cells (Mzb1+/Ighg1+). **C** Pseudotime analysis diagram of microglia in the gliosis zone of childhood ACP tissue. Monocle2 software was used to perform pseudotime analysis of the cell clusters annotated as microglia (clusters 2, 3, and 7), and the starting point of the cells was calculated with Monocle software. Microglia can gradually differentiate from cluster 7 into cluster 2 and cluster 3. **D** scVelo software was used to analyze the RNA rate of the cell population (clusters 2, 3, and 7) annotated as microglia and simulate the differentiation direction map of a single cell. **E** The differentialGeneTest function of monocle2 was used to find genes with expression patterns that changed over pseudotime, and a heatmap of the obtained differential gene list was drawn (qval < 1e-4). The abscissa is the time value of the pseudotime trajectory, as the time pass (arrow pointed), the microglia showed inflammation activation, increased synthesis of proinflammatory factors, activation of inflammation-related pathways, and increased response to beta-amyloid. **F** The expression of the MHC-II, APOE, and CD74 was gradually increased during the differentiation of microglia. **G** Interaction analysis between the microglial cell population (csf1r+/cx3cr1+) and cluster 5 (snap25+/syt1+), which was identified as neurons. The intercellular interaction of CD74–APP and CD74–COPA was significantly enhanced between microglia in the activated state of inflammation (cluster 2 and cluster 3) and neurons. In the interaction, microglia express the ligand gene CD74, and neurons express the receptor genes APP and COPA

**Fig. 6:**
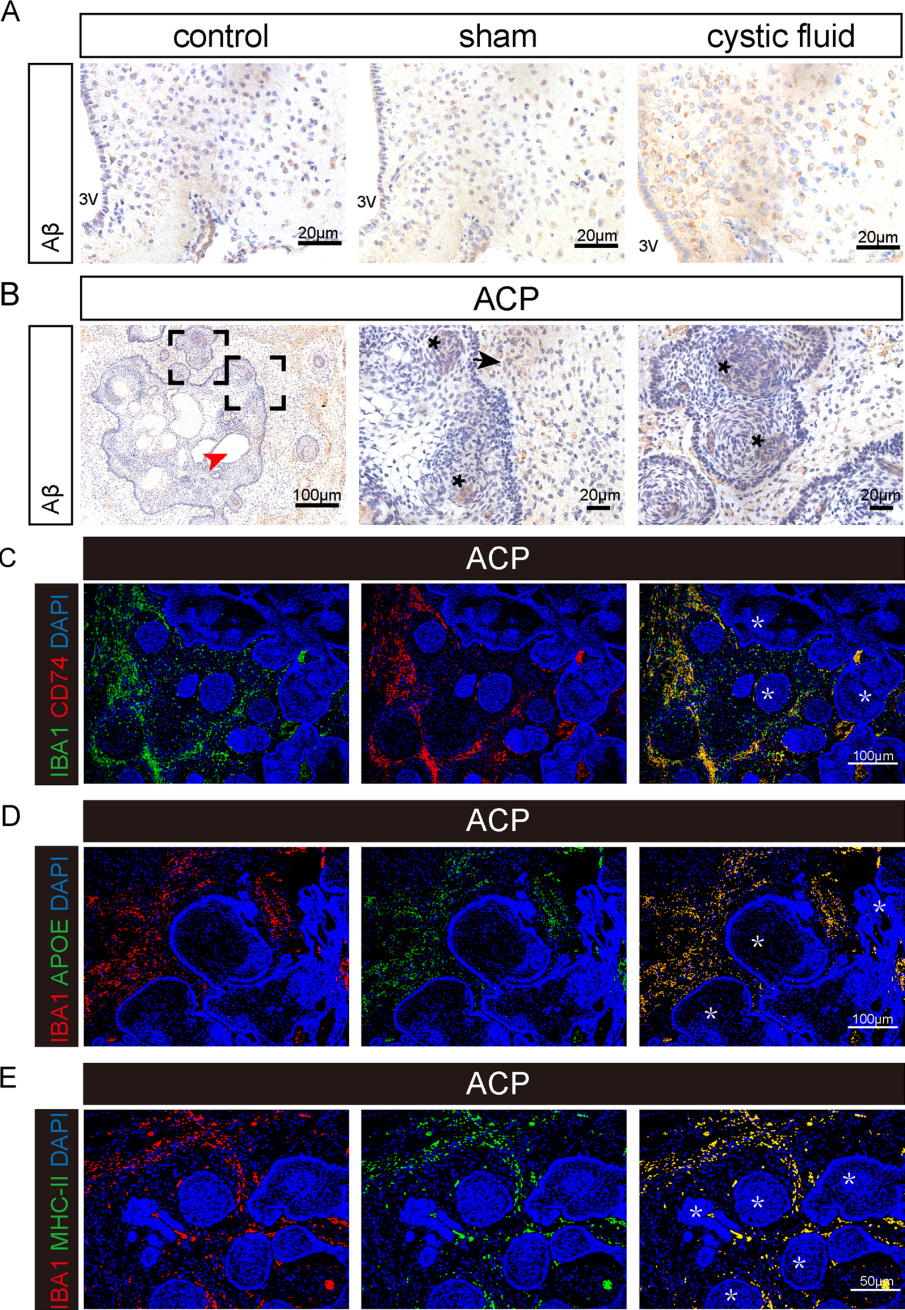
Expression of pathological markers of neurodegenerative diseases on mouse hypothalamus and childhood ACP tissue. **A** The results of immunohistochemistry. Amyloid-β (Aβ) is highly expressed in the mouse hypothalamus ARC after injection of ACP cystic fluid (3 V: third ventricle). **B** The results of immunohistochemistry. Amyloid-β (Aβ) is deposited in the whorl-like cells and in the gliosis zone of childhood ACP tissues. The red arrow indicates the cystic structure. The asterisk indicates that Aβ was deposited in whorl-like cells, and the black arrow indicates that Aβ was deposited in the gliosis zone. **C**–**E** Immunofluorescence results. Expression of CD74, APOE, and MHC-II in microglia in the gliosis zone of ACP. Asterisks indicate tumor tissue, Iba1 indicates microglia, and DAPI indicates nuclei

To obtain the ACP single-cell gene expression profile of clinical samples, we collected and pretreated childhood ACP specimens obtained during the operation according to the procedures described in the methods and then performed single-cell RNA sequencing. After filtering low-quality samples and cells, 6632 cells were obtained. The median UMI was 4653.5/cell, and the median number of genes per cell was 2106/cell. As a result, 35954 genes were detected in all cells. After removing the mitochondrial genes from all the cells through an unsupervised strategy, clustering was performed, and 16 populations were found. Through the expression of markers expressed by some specific cell clusters [42–46], we determined the cell type represented by each cluster and identified a large number of glial cells, including microglia (Additional file 4: Fig. S4C). The remaining immune cells included B plasma cells and T cells, and because the growth pattern of this tumor sample is closely related to the anatomical location of the hypothalamus and third ventricle, a small number of neurons were been identified (Fig. 5A, B, Additional file 1: Fig. S1B). By referencing previous studies [55–57], we determined that cluster 0 and cluster 11 are ACP cells (TP63+), and GO enrichment analysis of all genes in these two groups of cells suggested that they are related to osteogenesis and calcification, as well as activation of the Wnt pathway (Fig. 5B, Additional file 4: Fig. S4A, B), which allowed us to determine the identities of these two groups of cells. As mentioned above, Monocle2 software was used to analyze the cell populations (clusters 2, 3, and 7) annotated as microglia, and the starting point of the cells was calculated with Monocle software. Microglia can gradually differentiate from cluster 7 into cluster 2 and cluster 3 (Fig. 5C). At the same time, scVelo software was used to analyze the RNA rate of the cell populations annotated as microglia (clusters 2, 3, and 7) and simulate the differentiation direction map of a single cell (Fig. 5D). The differentialGeneTest function was used in monocle2 to find genes with expression patterns that changed over pseudotime and to draw a heatmap of the obtained differential gene list (qval<1e-4). The abscissa is the pseudotime trajectory value. The graph shows the change process of key genes during cell state change. At the same time, we performed GO enrichment analysis on the three clusters in the heatmap one by one. The analysis results included inflammation activation, increased synthesis of proinflammatory factors, activation of inflammation-related pathways, and increased beta-amyloid response (Fig. 5E). This suggests that the microglia present in childhood ACP are in the same state as the abovementioned mouse hypothalamic microglia, which undergo inflammatory activation in response to cyst fluid. In addition, the expression of the MHC-II gene (homologous to the mouse H-2 gene), APOE, and CD74 genes also gradually increased during the differentiation process of microglia in childhood ACP tissue (Fig. 5F). Finally, we analyzed the cell–cell interaction between the microglial cell population (csf1r+/cx3cr1+) and cluster 5 cells identified as neurons (snap25+/syt1+), and it was found that cluster 2 and cluster 3 microglia, under an inflammatory activated state, and neurons also have the same cell–cell interaction relationship between CD74–APP and CD74–COPA that was observed in the hypothalamus of mice injected with ACP cyst fluid (Fig. 5G). Because there are very few neurons in ACP specimens, their subtypes are difficult to confirm. To further verify the above results, we tested MHC-II, APOE, CD74, APP, and Aβ in ACP tumor tissues and found MHC-II, APOE, and CD74 expression on microglia (IBA1+) in the ACP nerve junction area (Fig. 6C–E), suggesting that these microglia are also in the same state of inflammatory activation. Interestingly, we also found the expression of Aβ in the whorl-like cells and the gliosis zone of ACP tumor tissue (Fig. 6B). These results suggest that pathological changes similar to neurodegenerative diseases may have also occurred in the ACP tumor microenvironment.

## Discussion

Although ACP is a benign tumor, the poor quality of life caused by hypothalamic injury has been difficult to solve [3]. The hypothalamus, a center that maintains hormone balance, energy metabolism, and homeostasis, is closely adjacent to the ACP, making it the most susceptible structure to be violated by the ACP, causing hormonal, metabolic, and electrolyte disorders and leading to lifelong medical care for patients [58]. Although the mainstream view is that the tumor compression and surgical trauma cause damage to the hypothalamus [59, 60], the effect of cyst fluid, a unique component of ACP, on the hypothalamus is unknown. To eliminate the interference of surgical trauma and tumor compression, we pushed the tip of the microinjector into the upper part of the bilateral hypothalamus of the mouse and then slowly injected the cyst fluid or PBS to avoid mechanical damage as much as possible to allow the fluid to penetrate into the hypothalamus. After 8 weeks, the mice in cystic fluid group showed growth retardation and obesity tendency, and the decrease in serum GH and GHRH and the short-term increase in food intake were corresponded to the appropriate phenotype. Growth retardation and obesity are the most intuitive manifestations of hypothalamic injury [58]. In the rat hypothalamic electrical damage model [61], the phenotypes of shorter body length, abnormal obesity, and continuous increased food intake implying that although the damage caused by ACP cystic fluid in the hypothalamus is not as strong as electrical damage, it still has a deleterious effect on the hypothalamus. ARC is a nucleus that regulates energy metabolism and body growth [12, 13]. We found that the expression of the Fgfr2 and Rnpc3 genes was significantly decreased in the ARC neurons of mice in the cystic fluid group. Their knockout and mutation caused dwarfism in mice and humans [25–28]. At the same time, the Npy and Pcsk1n genes were significantly highly expressed in ARC neurons in the cyst fluid group. In addition, ACP cystic fluid caused high expression of the Sst gene in the mouse hypothalamus, and the growth inhibitory hormone encoded by Sst antagonizes growth hormone [12] and the activation of Sst neurons promote food intake and obesity [49]. Pcsk1n is an endogenous inhibitor of Pcsk1. The high expression of proSAAS encoded by Pcsk1n causes obesity and diabetes in mice [29]. Pcsk1 gene mutations are common in obese human populations [30, 32]. The significant changes in the expression of the above genes in ARC neurons of mice in the cystic fluid group may help explain the growth retardation and increased obesity index observed in mice and provide new target genes for the further study of ACP. The hypothalamic orexin system includes Npy/Agrp, Pomc, Mc4r, and Mc3r [36]. Pomc inhibits food intake by exciting Mc4r and Mc3r; in contrast, Npy/Agrp promotes food intake by inhibiting Mc4r and Mc3r [35]. Npy/Agrp and Pomc maintain the balance of energy metabolism through Mc4r and Mc3r [34, 36]. The significant enhancement of the Agrp–Mc3r interaction between Npy/Agrp neurons and Ghrh neurons seems to imply that ACP cystic fluid disrupts the orexin system in the hypothalamus.

As previously shown, ACP cystic fluid has a damaging effect on neurons in vitro [24] ; the abovementioned gene changes may be caused by the direct damage of cystic fluid to ARC neurons. However, through pseudochronological analysis and GO pathway enrichment analysis, we found that ACP cystic fluid caused inflammatory activation of microglia and increased expression of IL-6, which was also verified in PCR experiments. Pseudochronological analysis suggested that activated microglia mainly exhibit inflammation activation, increased synthesis of proinflammatory factors, activation of inflammation-related pathways, increased response to lipoprotein particle stimulation, and beta-amyloid (Aβ) synthesis and response. The signal that positively regulates neuronal apoptosis is significantly strengthened in ARC neurons, which is similar to the results of in vitro experiments previously reported by Ghosh et al. [24]. It is worth noting that beta-amyloid (Aβ) produced by APP, a pathological sign of Alzheimer's disease, causes inflammatory damage to neurons and is responsible for the degeneration of synapses and the apoptosis of neurons [38]. In a study of Alzheimer’s disease (AD), authors discovered the cell interaction between CD74 and APP for the first time and proposed that the high expression of CD74 in the brains of AD patients may be caused by the overexpression of APP in AD patients, which may contribute to a biological negative feedback mechanism to resist the upregulation of harmful APP processing [37]. This hypothesis suggests that the CD74–APP interaction is a way for the body to fight neurodegenerative diseases. In this study, the significantly enhanced CD74–APP interaction between inflammatory activated microglia and ARC neurons in the mouse hypothalamus suggested that ACP cystic fluid may cause neurodegenerative lesions in the hypothalamus. The COPI coat plays a role in the processing and transport of amyloid precursor protein (APP), and α-COP (COPA) is a member of the heptameric COPI outer shell complex. Studies have shown that knocking down COPA inhibits the maturation and cleavage of APP, resulting in a decrease in the release of Aβ, and the high expression of COPA promotes the release of Aβ, increasing the toxic effect on neurons [54]. In our study, the high expression of Aβ and the enhanced interaction of CD74–COPA in the mouse hypothalamus further suggested that ACP cystic fluid may cause pathological changes similar to those observed in neurodegenerative diseases.

In ACP tumor tissues, we also verified the same phenomenon of inflammatory activation of microglia observed in mice. Activated microglia also showed inflammatory activation, increased synthesis of proinflammatory factors, activation of inflammation-related pathways, increased response to lipoprotein granule stimulation, and increased synthesis and response of beta-amyloid (Aβ). Interestingly, in ACP tumor tissues, we found that CD74–APP and CD74–COPA interactions were also significantly strengthened between inflammatory activated microglia and neurons. CD74–APP, an intercellular interaction that is unique to Alzheimer's disease [37], was discovered for the first time in ACP tumor tissue. Although there are some gliosis bands in ACP tumor tissues, there is very little nerve tissue, so single-cell RNA sequencing cannot identify the specific types of neurons identified. The expression of MHC-II, APOE, and CD74 was also found on microglia (IBA1+) in the ACP nerve junction area. The expression of Aβ, the pathological marker of Alzheimer's disease [38], in the whorl-like cells and gliosis zone of ACP tumor tissues suggests that a pathological change similar to neurodegenerative disease may also occur in the ACP tumor microenvironment. This is the first time that Aβ has been discovered in ACP and provides a new research direction for further study of ACP.

In addition, the single-cell RNA sequencing data indicated that the expression of CD68, an index of phagocytosis of microglia [62], was increased in cluster 7 microglia in the cystic fluid group, and this was verified in tissue samples of ACP and hypothalamic tissues of mice. ACP cyst fluid is rich in cholesterol and other lipid particles. In the hypothalamus, these foreign bodies inevitably recruit a large number of immune cells for elimination. The pseudochronological analysis and GO enrichment analysis indicated that the expression of APOE in inflammation activated microglia was enhanced, and lipoprotein granule responses and Myd88-TLR-related pathway responses were also enhanced. We speculate that microglia can clear lipids in cyst fluid while activating inflammation-related pathways. Therefore, if we can control inflammation while enhancing the "noninflammatory phagocytosis" of microglia, it may be possible to inhibit the occurrence of hypothalamic-related inflammation caused by ACP cyst fluid. Notably, the senescent cells, which secrete SASPs (senescence-associated secretory phenotypes), including IL-6, are present in the ACP tumors [63]. Martinez et al. [64] found that SASPs play a critical role in the initiation of the ACP and promote inflammation in the nerve tissues surrounding tumors via the paracrine function. At the same time, the SASPs are associated with the pathogenesis of the neurodegenerative diseases [65] ; therefore, the influences of the senescent cells in the ACP tumors on the microglia activation and on the Aβ deposition could not be excluded.

The ACP cystic fluid we injected into the hypothalamus of mice is present only temporarily. Although we simulated the leakage process of cyst fluid during ACP resection, Aβ, observed in the mouse hypothalamus injected with ACP cystic fluid, is also expressed in whorl-like cells and gliotic tissues of ACP tumors. In current studies, no Aβ was found in the analysis of the composition of ACP cystic fluid [21, 66], which implies that the leakage of cystic fluid not only occurs during surgical resection but also may occur slowly during prolonged disease progression and lead to the production of Aβ. Interestingly, our team previously found that the key protein PPAR-γ [15], induces adipogenic differentiation and promotes the formation of ACP cyst fluid, is also expressed in whorl-like cells. It was found that APOE uses cholesterol to transport neuronal amyloid precursor protein (APP) in and out of lipid clusters to promote the production of Aβ [67]. In our experiment, the expression of APOE was upregulated in microglia both in ACP gliosis tissue and the hypothalamus of mice injected with ACP cystic fluid. This seems to imply that the lipid component (cholesterol) in cystic fluid is the key factor leading to the deposition of Aβ.

In short, we have an unprecedented understanding of ACP cystic fluid. Avoiding leakage of cystic fluid during surgical resection is a good measure to protect the hypothalamus from hormonal and energy metabolism disorders. The animal model established in this study is helpful for the research and treatment of ACP-related hypothalamic injury. At the same time, the Npy, Fgfr2, Rnpc3, Sst, and Pcsk1n genes, and the cell-to-cell interactions CD74–APP, CD74–COPA, and Agrp–Mc3r, and Aβ may provide potential targets for further study of ACP. In this study, a pathological phenomenon very similar to neurodegenerative diseases was found in the ACP tumor tissue, implying that medicines used to treat neurodegenerative diseases may help prevent ACP damage to the hypothalamus.

## Conclusion

In this study, a novel animal model of ACP cystic fluid-hypothalamic injury was established. For the first time, it was found that ACP cystic fluid affects the expression of the Npy, Fgfr2, Rnpc3, Sst, and Pcsk1n genes and the cellular interaction of Agrp–Mc3r in hypothalamic neurons and can trigger inflammatory activation of microglia to damage the hypothalamus, which may be related to the upregulation of the CD74–APP interaction and deposition of β-amyloid, implying that there may be a similar mechanism between ACP cystic fluid damage to the hypothalamus and neurodegenerative diseases.

## Limitations

This research is limited by the inherent limitation related to the depth of sequencing that plagues all mononuclear and single-cell RNA-seq research: changes in the expression of low-abundance transcripts cannot be detected. The current sample size was chosen for pragmatic reasons, including cost. Although the sample size was small, we verified the finding through a variety of experimental methods and obtained similar results. As the cost of single-cell RNA-seq research decreases, future research may verify our findings in a large sample.

## Supplementary Information

**Additional file 1:** **Figure S1**. **A**. Schematic diagram of mouse stereotactic surgery. The injection coordinates were 1.80 mm behind the bregma, 0.35 mm on both sides of the sagittal sinus, and 5.00 mm from the brain surface. **B**. In each subcluster of cluster 3 of mice from the cystic fluid group, the pathways related to APP synthesis and feeding behavior were more significantly upregulated than in the subclusters of cluster 3 of mice from the sham operation group. Subcluster 3-0 mainly upregulated pathways related to APP synthesis and neuronal apoptosis, subcluster 3-1 and subcluster 3-2 mainly upregulated pathways related to APP synthesis, and subgroups 3-3 and 3-4 mainly upregulated pathways related to feeding. **C**. The results of immunohistochemistry. The expression of Npy in the hypothalamus of mice in the cystic fluid group was upregulated, and the number of Npy+ cells in the ARC also increased. 3V: The third ventricle. The needle track (*indication) can be seen above the third ventricle in the cystic fluid group and the sham operation group. **D**. PCR detection results. The expression of the Sst gene and Pcsk1n gene was significantly upregulated, and the expression of the Fgfr2 and Rnpc3 genes was significantly downregulated in the hypothalamus of mice in the cystic fluid group. **E**. The ratio of cells in the cystic fluid group and the sham operation group detected by single-cell RNA sequencing. Cluster 11 (microglia activated by inflammation) was mainly contributed by the cystic fluid group. The high proportions of meningeal cells (clusters 14 and 16) in the cystic fluid group was caused by the incorporation of meningeal tissue during the collection of the mouse hypothalamus, which did not affect the experimental results.

**Additional file 2:** **Figure S2**. **A**. ACP cystic fluid activates mouse microglial BV2 cells in in vitro experiments. The activated microglia showed amebic-like changes: enlarged cell bodies, short axons, and increased numbers of axons. **B**. Single-cell RNA sequencing detected a significant upregulation of CD68 expression in cluster 7 (microglia) in the cyst fluid group. **C**. PCR results showed that the expression of IL-6 and CD68 in the hypothalamus of mice in the cystic fluid group was significantly upregulated. **D**. The results of immunohistochemistry showed that the expression of CD68 in the hypothalamus of mice in the cystic fluid group was upregulated. 3V: The third ventricle. The needle track (*indication) can be seen above the third ventricle in the cystic fluid group and the sham operation group. **E**. The immunofluorescence results showed the expression of CD68 in microglia in the gliosis zone of childhood ACP. Iba1 indicates microglia, DAPI indicates nuclei, and asterisks indicate tumor tissue.

**Additional file 3: Figure S3**. Single-cell RNA sequencing showed the cell interaction between each subcluster of cluster 3 in the cystic fluid group and the sham operation group. T: cystic fluid, H: sham. Agrp–Mc3r was significantly strengthened between subcluster 3-3 (Agrp/Npy neurons) and subcluster 3-4 (Ghrh neurons) in the cystic fluid group. In the interaction relationship, subclusters 3-3 express the ligand gene Agrp, and subclusters 3-4 express the receptor gene Mc3r.

**Additional file 4: Figure S4**. **A, B**. Single-cell RNA sequencing showed that in childhood ACP tissues with gliosis, Wnt pathways and pathways related to ossification, osteoblast differentiation, and chondrocyte differentiation and development were specifically and highly expressed in cluster 0 and cluster 11, further clarifying that they are ACP cells. **C**. Single-cell RNA sequencing showed the cell ratio of each cluster in childhood ACP tissue. In addition to ACP cells, microglia, T cells and B cells and a very small number of neurons are present.

**Additional file 5.**The details for materials and methods.

**Additional file 6.**The details for ScRNA-seq data analysis.

## Data Availability

All data generated and/or analyzed during the current study are available from the corresponding author on reasonable request.

## Abbreviations

ACP: Adamantinomatous Craniopharyngioma
ARC: Arcuate Nucleus
ScRNA-seq: Single-cell RNA sequencing
GO: Gene Ontology
APP: Amyloid Precursor Protein
Aβ: β-Amyloid
Npy: Neuropeptide Y
Agrp: Agouti-related peptide
Mc3r: Melanocortin-3 receptor
GH: Growth Hormone
GHRH: Growth Hormone-Releasing Hormone
PBS: Phosphate-Buffered Saline
