# Supplementary figures and images for: Adamantinomatous craniopharyngioma cyst fluid can trigger inflammatory activation of microglia to damage the hypothalamic neurons by inducing the production of β-amyloid
Source: J Neuroinflammation. 2022 May 7;19:108. doi: 10.1186/s12974-022-02470-6 (PMC9080190; doi:10.1186/s12974-022-02470-6)

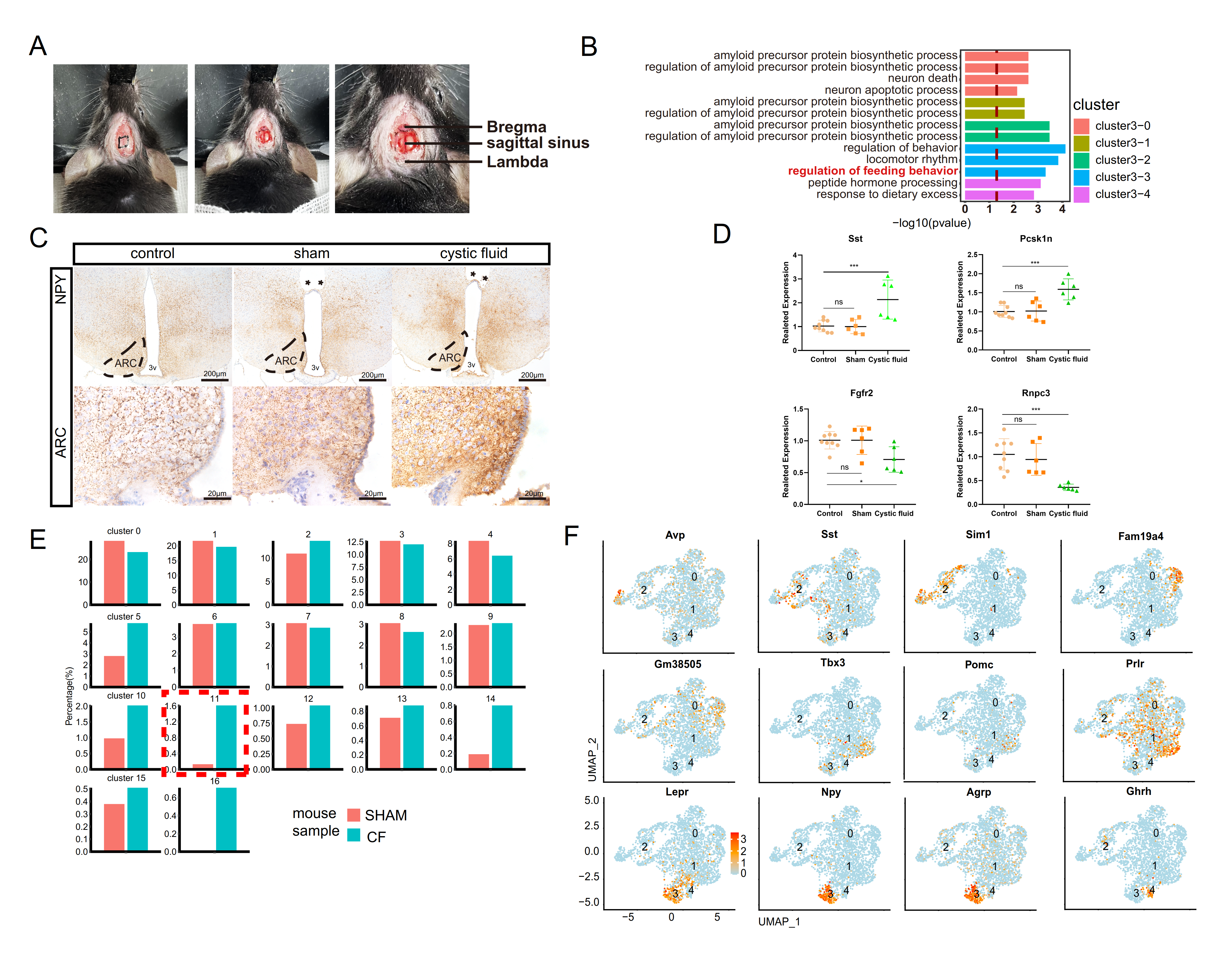

Supplement: Supplementary file 1 — Additional file 1: Figure S1. A. Schematic diagram of mouse stereotactic surgery. The injection coordinates were 1.80 mm behind the bregma, 0.35 mm on both sides of the sagittal sinus, and 5.00 mm from the brain surface. B. In each subcluster of cluster 3 of mice from the cystic fluid group, the pathways related to APP synthesis and feeding behavior were more significantly upregulated than in the subclusters of cluster 3 of mice from the sham operation group. Subcluster 3-0 mainly upregulated pathways related to APP synthesis and neuronal apoptosis, subcluster 3-1 and subcluster 3-2 mainly upregulated pathways related to APP synthesis, and subgroups 3-3 and 3-4 mainly upregulated pathways related to feeding. C. The results of immunohistochemistry. The expression of Npy in the hypothalamus of mice in the cystic fluid group was upregulated, and the number of Npy+ cells in the ARC also increased. 3V: The third ventricle. The needle track (*indication) can be seen above the third ventricle in the cystic fluid group and the sham operation group. D. PCR detection results. The expression of the Sst gene and Pcsk1n gene was significantly upregulated, and the expression of the Fgfr2 and Rnpc3 genes was significantly downregulated in the hypothalamus of mice in the cystic fluid group. E. The ratio of cells in the cystic fluid group and the sham operation group detected by single-cell RNA sequencing. Cluster 11 (microglia activated by inflammation) was mainly contributed by the cystic fluid group. The high proportions of meningeal cells (clusters 14 and 16) in the cystic fluid group was caused by the incorporation of meningeal tissue during the collection of the mouse hypothalamus, which did not affect the experimental results. [file 12974_2022_2470_MOESM1_ESM.tif]

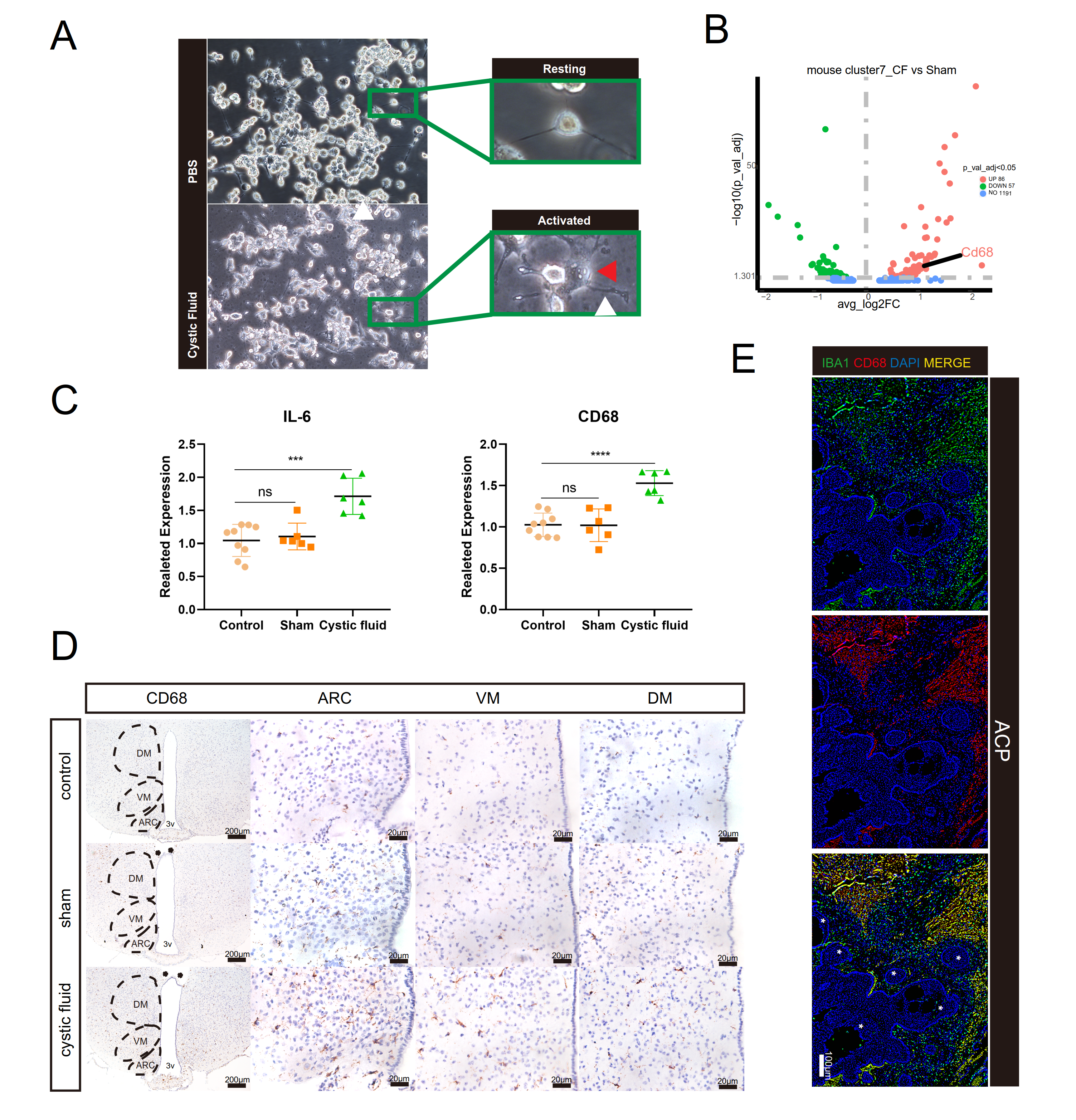

Supplement: Supplementary file 2 — Additional file 2: Figure S2. A. ACP cystic fluid activates mouse microglial BV2 cells in in vitro experiments. The activated microglia showed amebic-like changes: enlarged cell bodies, short axons, and increased numbers of axons. B. Single-cell RNA sequencing detected a significant upregulation of CD68 expression in cluster 7 (microglia) in the cyst fluid group. C. PCR results showed that the expression of IL-6 and CD68 in the hypothalamus of mice in the cystic fluid group was significantly upregulated. D. The results of immunohistochemistry showed that the expression of CD68 in the hypothalamus of mice in the cystic fluid group was upregulated. 3V: The third ventricle. The needle track (*indication) can be seen above the third ventricle in the cystic fluid group and the sham operation group. E. The immunofluorescence results showed the expression of CD68 in microglia in the gliosis zone of childhood ACP. Iba1 indicates microglia, DAPI indicates nuclei, and asterisks indicate tumor tissue. [file 12974_2022_2470_MOESM2_ESM.tif]

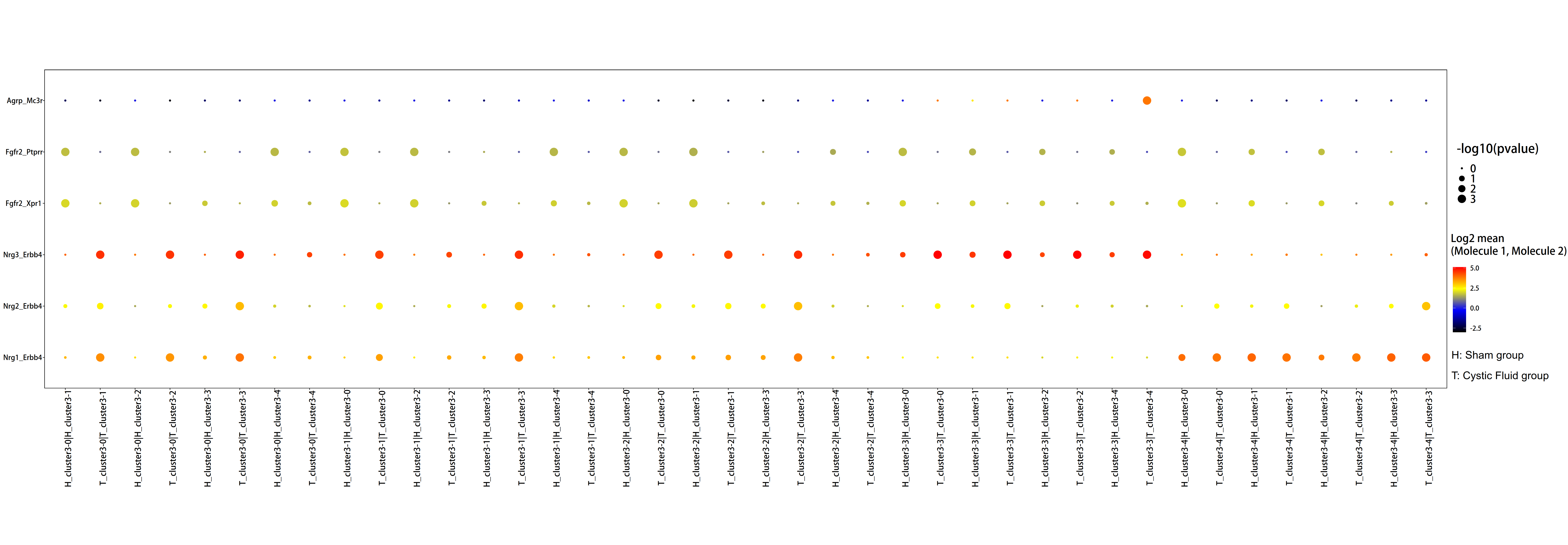

Supplement: Supplementary file 3 — Additional file 3: Figure S3. Single-cell RNA sequencing showed the cell interaction between each subcluster of cluster 3 in the cystic fluid group and the sham operation group. T: cystic fluid, H: sham. Agrp–Mc3r was significantly strengthened between subcluster 3-3 (Agrp/Npy neurons) and subcluster 3-4 (Ghrh neurons) in the cystic fluid group. In the interaction relationship, subclusters 3-3 express the ligand gene Agrp, and subclusters 3-4 express the receptor gene Mc3r. [file 12974_2022_2470_MOESM3_ESM.tif]

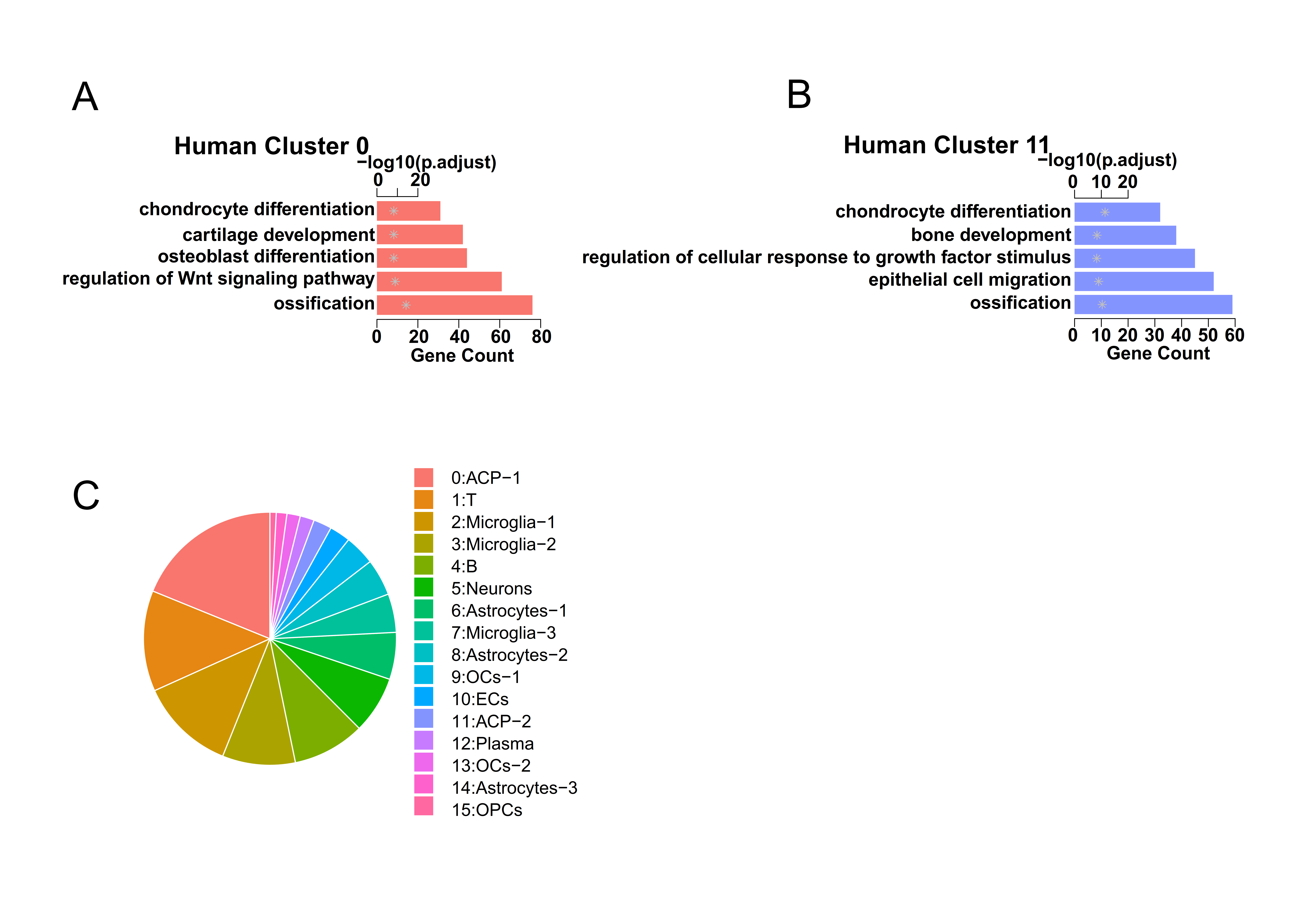

Supplement: Supplementary file 4 — Additional file 4: Figure S4. A, B. Single-cell RNA sequencing showed that in childhood ACP tissues with gliosis, Wnt pathways and pathways related to ossification, osteoblast differentiation, and chondrocyte differentiation and development were specifically and highly expressed in cluster 0 and cluster 11, further clarifying that they are ACP cells. C. Single-cell RNA sequencing showed the cell ratio of each cluster in childhood ACP tissue. In addition to ACP cells, microglia, T cells and B cells and a very small number of neurons are present. [file 12974_2022_2470_MOESM4_ESM.tif]
