# Supplementary material for: Adamantinomatous craniopharyngioma cyst fluid can trigger inflammatory activation of microglia to damage the hypothalamic neurons by inducing the production of β-amyloid
Source: J Neuroinflammation. 2022 May 7;19:108. doi: 10.1186/s12974-022-02470-6 (PMC9080190; doi:10.1186/s12974-022-02470-6)
Supplement: Supplementary file 5 — Additional file 5.The details for materials and methods. [file 12974_2022_2470_MOESM5_ESM.docx]

**Materials and Methods**

**1. Animals:** Pregnant C57/BL/J6 female mice were purchased from the Experimental Animal Center of Nanfang Hospital, Southern Medical University, and one mouse was placed in each cage. The pups were weaned 3 weeks after birth, and the male pups were selected and kept in cages with 4-6 pups each. The animals were maintained in an environment with a 12/12-hour light/dark cycle (07:00-19:00 light/19:00-7:00 dark), a controlled temperature of 20-22°C, a humidity level of 50%-55%, with free access to normal food and water. We screened young (6 weeks old) male mice weighing 19-20 g as experimental subjects. We selected mice of this weight and age because they would be able to tolerate brain stereotactic surgery in our preliminary experiments, and they would continue to grow, which would help us observe the changes in their growth process and outcomes. Mice were randomly divided into 3 groups, and each group contains 8 mice .The mice in the cystic fluid group (n=8) and the sham operation group (n=8) were received stereotactic surgery , but the mice in the control group(n=8) did not receive any intervention .After stereotactic surgery, the mice were placed in the same environment as before the operation and fed normal food and water. The average food intake, average water intake, and average weight of each group of the mice were recorded every 3 days, and the body length (distance from the tip of the nose to the anus) was recorded every 7 days. The above indicators did not change from the 7th week after the operation, so we extended the observation period by another week; that is, the above indicators were recorded to 8 weeks after the operation, and the rodent obesity formula was used to calculate the obesity index (Lee index: body weight^1/3^ divided by body length).

All operations were approved by the Laboratory Animal Center and the Ethics Committee of Southern Medical University and complied with the National Institute of Health Guidelines for the Protection and Use of Laboratory Animals.

**2. Collection of childhood ACP cystic fluid and tumor tissue:** We screened patients with childhood ACP (5-12 years old) for tissue that infringed on the hypothalamus and collected ACP cystic fluid. These children received tumor resection and surgery at the Neurosurgery Department of Southern Hospital of Southern Medical University. The ACP cystic fluid was collected with a syringe during the surgery, and the cerebrospinal fluid and blood were not mixed. The excised ACP tumor tissue included the gliosis zone. The patients’ parents all signed an informed consent form expressing their willingness to allow the use of the excised tumor tissue and cystic fluid for scientific research, and the study received approval from the Ethics Committee of Southern Medical University. The collected tumor tissue and cystic fluid from surgery were immediately stored in a freezer at -80°C. Finally, we collected tumor tissues and cystic fluid from 8 pediatric patients.

**3. Stereotactic surgery:** The mice were anesthetized by inhalation with isoflurane (2%, 2 L O2/min), and the head was fixed with a stereotaxic instrument (Reward, Shanghai, China). The bregma and lambda were exposed, and a microsyringe (Gao Ge, Shanghai, China) was fixed on the stereotaxic instrument. Stereotactic surgery simulates the leakage of cystic fluid during ACP resection. Therefore, to avoid mechanical damage to the hypothalamus, we advanced the tip of the microinjector to just above the hypothalamus and then slowly injected the ACP cystic fluid. To allow the ACP cystic fluid to penetrate into the hypothalamus more thoroughly, it was maintained for 5 minutes after each injection of 1 µl cystic fluid. To prevent reflux, the microsyringe was held in place for 10 minutes and then slowly withdrawn. According to the sixth edition of the Mouse Brain Anatomy Atlas, the injection coordinates were as follows: 1.80 mm from bregma to the caudal side, 0.35 mm from both sides of the midline, and 5.00 mm from the brain surface. The skull was milled out in the target area, the microsyringe was filled with 20 µl of cystic fluid or PBS(Phosphate Buffered Solution), and 10 µl was injected into the hypothalamus on each side. Cystic fluid group: ACP cystic fluid was injected into the bilateral hypothalamus. Sham operation group: Same amount of PBS was used to inject into the bilateral hypothalamus . Control group: No treatment was administered. Each group included 8 mice, and each mouse in the cystic fluid group was injected with cystic fluid from a different patient.

**4. Single-cell RNA sequencing:** Eight weeks after the operation, a mouse was randomly selected from the cystic fluid group and the sham operation group respectively. After anesthetization, their hypothalamus were removed immediately under a light microscope for single-cell RNA sequencing. At the same time, a sample of fresh childhood ACP tumor tissue (with gliosis zone) immediately after collection during clinical surgery was also used for single-cell RNA sequencing. Details are provided in supplementary file 6.

**5. Immunohistochemistry and immunofluorescence:** Eight weeks after surgery, 4 mice were randomly selected from the control group, the cystic fluid group and the sham operation group respectively ,the mice were anesthetized and perfused transcardially with saline, followed by internal fixation with 4% paraformaldehyde in PBS (phosphate-buffered saline, pH=7). The brain was removed and soaked with 4% formaldehyde for 48 hours. The entire hypothalamus was coronally cut out with stainless steel brain matrices (BB-NMJ), dehydrated, and then embedded in paraffin, and then 2 µm sections were made. Eight case of the ACP tumor tissues were also soaked with 4% formaldehyde for 48 hours, dehydrated, and then embedded in paraffin, and then 2 µm sections were made . After baking in a 60°C oven for 12 hours, the samples were dewaxed with xylene and alcohol (100%-70%), washed with PBS (pH=7) and EDTA (pH=9) for antigen retrieval, cooled at room temperature for 2 hours after antigen retrieval, washed with PBS, then blocked with 3% hydrogen peroxide for 10 minutes. After washing with PBS, the cells were blocked with 10% goat serum for 1 hour. Sections were incubated with primary antibody (**immunohistochemistry:** rabbit anti-mouse/human beta amyloid antibody, Proteintech, USA, 25524-1-AP, dilution 1:200; rabbit anti-mouse CD68 antibody, Cell Signaling Technology, USA, #97778, dilution 1:200; rabbit anti-mouse neuropeptide Y antibody, Cell Signaling Technology, USA, #11976, dilution 1:200). **Immunofluorescence:** A rabbit anti-human CD68 antibody (ABcam, China, ab213363, dilution 1:100); goat anti- human Iba-1 antibody (ABcam, China, ab5076, dilution 1:100); rabbit anti-human CD74 antibody (Cell Signaling Technology, USA, #77274, dilution 1:200); mouse anti-human MHC Class II antibody (Cell Signaling Technology, USA, #68258, dilution 1:100); and mouse anti-human APOE antibody (Proteintech, USA, 66830-1-Ig, dilution 1:200)) at 4°C overnight. The immunohistochemical sections were washed with PBS and then incubated with secondary antibody (universal enzyme-labeled goat anti-mouse/rabbit IgG polymer, ready-to-use, PV-6000, ZSGB-BIO) for 1 hour at room temperature. The immunofluorescence sections were protected from light and incubated with fluorescent secondary antibody (donkey anti-rabbit IgG, Alexa Fluor® 555, ABcam, ab150074, dilution 1:1000; rabbit anti-goat IgG, Alexa Fluor® 488, ABcam, ab150141, dilution 1:1000; goat anti-mouse IgG, Alexa Fluor® 555, ABcam, ab150141, dilution 1:1000) at room temperature for 1 hour. The sections were observed and images were captured under an Olympus microscope (Olympus BX63, Japan).

**6. ELISA test for plasma hormone levels:** Eight weeks after the operation, the mice were anesthetized with isoflurane, and then 600 µl of blood was drawn from the tail veins into an anticoagulation centrifuge tube, placed at 4°C for 3 hours, and stratified and centrifuged at 4°C for 20 minutes (3000 rpm). The supernatant was collected as the serum, and each test sample was 50 µl of serum. Serum was used immediately for ELISA. Samples were diluted and subjected to standard ELISA to determine GH and GHRH levels in accordance with the manufacturer's protocol (mouse GH ELISA kit, EZRMGH-45K; mouse GHRH ELISA kit, ELK2562; Millipore, Billerica, MA, USA).

**7. Mouse microglia (BV2) were stimulated with cystic fluid in vitro.** In vitro, cystic fluid and culture medium at a ratio of 1:20 was prepared, in which mouse microglia (BV2) were cultured for 24 hours at 37℃.The control group of BV2 cells was cultured in saline-mixed culture medium (saline:culture medium=1:20) for 24 hours at 37℃.

**8. RT q-PCR test:** Eight weeks after surgery, 4 mice from the control group, 3 mice from the cystic fluid group and 3 mice from the sham operation group were anesthetized .Total RNA was extracted from the mouse hypothalamus using RNAiso Plus reagent (9108, TaKaRa Bio Inc., Tokyo, Japan) according to the manufacturer's instructions. The quantity and concentration of RNA were assessed by measuring absorbance with a spectrophotometer at A260/280. The [reverse transcription](https://www.sciencedirect.com/topics/medicine-and-dentistry/reverse-transcription) reactions were performed using a PrimeScript RT reagent kit (RR047A; TaKaRa Bio Inc., Tokyo, Japan). RT–qPCR was performed with a SYBR Premix Ex Taq kit (RR420A; TaKaRa Bio Inc., Tokyo, Japan) on an ABI 7500 real-time PCR system (Applied Biosystems, Foster City, CA, USA) following the manufacturer's instructions. The relative expression levels of each sample were calculated using the 2^−ΔΔCt^ method with glyceraldehyde-3-phosphate dehydrogenase (GAPDH) as the endogenous control. Each experiment repeated was three times. The primer sequences used in qPCR were as follows: GAPDH forward: 5'-GGCACAGTCAAGGCTGAGAATG-3' and reverse: 5'-ATGGTGGTGAAGACGCCAGTA-3'; Mouse-IL-6 forward: 5'-CTCCCAACAGACCTGTCTATAC-3' and reverse: 5'-CCATTGCACAACTCTTTTCTCA-3'; Mouse-Sst forward: 5'-GAGCCCAACCAGACAGAGAATGATG-3' and reverse: 5'-TGCCATTGCTGGGTTCGAGTTG-3'; Mouse-Fgfr2 forward: 5'-CGCTTCATCTGCCTGGTCTTGG-3' and reverse: 5'-CGCTTCTCCATCTTCTCGGTGTTG-3'; Mouse-Rnpce forward: 5'-TGCACCCCTTCCACCTACATCC-3' and reverse: 5'-TCATTCTCTGTCGGTCCTCCTCATC-3'; Mouse-Pcsk1n forward: 5'-GCCGATCTGTGGACCAGGATTTG-3' and reverse: 5'-AGGGGTTCTCCAGGCGTTTGAC-3'; and Mouse-CD68 forward: 5'-CCTCTTGCTGCCTCTCATCATTGG-3' and reverse: 5'-GGCTGGTAGGTTGATTGTCGTCTG-3'.

**9. Statistical analysis :** All values are expressed as the mean±SEM and represent data from at least 3 repeated experiments. Data were analyzed using SPSS 22 (IBM, MIT) and GraphPad (San Diego, CA) software. If the mean satisfied the normal distribution, a parameter test was used, one-way ANOVA was used for data with a homogeneous variance, and Welch ANOVA was used for data with a heterogeneous variance. If the normal distribution was not satisfied, the Kraskal-Wall test was used. P < 0.05 (*), P < 0.01 (**), P < 0.001 (***) and P < 0.0001 (****) were considered to indicate statistical significance. NS(P > 0.05) means that there was no significant difference.
