# Supplementary material for: Adamantinomatous craniopharyngioma cyst fluid can trigger inflammatory activation of microglia to damage the hypothalamic neurons by inducing the production of β-amyloid
Source: J Neuroinflammation. 2022 May 7;19:108. doi: 10.1186/s12974-022-02470-6 (PMC9080190; doi:10.1186/s12974-022-02470-6)
Supplement: Supplementary file 6 — Additional file 6.The details for ScRNA-seq data analysis. [file 12974_2022_2470_MOESM6_ESM.docx]

### **Tissue prep, cDNA amplification, and library construction for 10× snRNA-seq**

The tissue was dissected out and flash frozen in liquid N2. Nuclei were isolated as followed by 10× Genomics demonstrate protocol (CG000375 • Rev A) .On the day of the experiment, frozen tissue was homogenized in NP40 Lysis Buffer with Protector RNAase Inhibitor (Sigma) and filtered through a 70μm strainer (falcon). Strained samples were centrifuged at 500 rcf × 5 min and pelleted nuclei were resuspended in wash buffer . Nuclei were strained again and recentrifuged at 500 rcf × 5 min. Washed nuclei were resuspended in wash buffer and stained nuclei underwent FACS sorting by 7-AAD (SML1633 sigma). Sorted nuclei were centrifuged at 500 rcf × 6 min and resuspended in wash buffer to obtain a concentration of 900 nuclei/μL. 15 µl of single-cell suspension at a concentration of ~900,000 cells/ml was loaded into one channel of the ChromiumTM Single Cell G Chip (10× Genomics, 1000120), aiming for a recovery of 8000–9000 cells. The Chromium Single Cell 3′ Library & Gel Bead Kit v3.1 (10× Genomics, 1000121) was used for single-cell barcoding, cDNA synthesis and library preparation, following manufacturer’s instructions according to the Single Cell 3′ Reagent Kits User Guide Version 3.1. Libraries were sequenced using the NovaSeq 6000 platform (Illumina) to a depth of approximately 300 million reads per library with 2×150 read length.

### **snRNA-seq data analysis**

**Alignment**

Raw reads of mouse were aligned to the mouse genome (mm10) and raw reads of human were aligned to the human genome (hg38), cells were called and gene expression matrices were generated for each sample by the cellranger (v6.0.1) count function with default parameters.

**Cell clustering and cell-type annotation**

The R package Seurat (v3.0.2) was used to cluster the cells in the merged matrix of mouse data. Cells with <800 transcripts and >4000 transcripts detected were first filtered out as low-quality cells. From the filtered cells, the gene expression matrices were normalized to the total UMI counts per cell and transformed to the natural log scale. To correct the batch effects, we integrated different samples using reciprocal PCA(rPCA) implemented in Seurat. We used the FindVariableFeatures function to obtain the top 2000 highly variable genes (HVGs) of each samples, and as the input dataset for batch effect correction. Using the FindIntegrationAnchors function the default dimensions (1:20), we found a set of pairwise correspondences between individual cells. These anchors are used for downstream integration of the objects. We used the IntegrateData function with the previously computed anchor set as a parameter to integrate all sample Seurat object. The default dimensions parameters (1:20) was used for the anchor-weighting procedure. The integrated dataset on all cells were then used to scale and center the genes, compute the principal components (PCs). After PCA to reduce dimensionality and build k-nearest neighbor graphs (k = 10) of the cells with the function FindNeighbors based on the Euclidean distance in the 50-dimensional PC space, the main cell cluster was identified using the Louvain-Jaccard graph-based method. For classifying all filtered cells, we set the clustering parameter resolution to 0.3 with the function FindClusters in Seurat. Next, the function RunUMAP with dimensions parameters (1:10) in Seurat was used to reduce high-dimension into two-dimension (2D) for visualization. Lastly, we run the Seurat FindAllMarkers function with the default parameters to identify the genes specifically expressed in each cluster. The significance of the differences in gene expression was determined using the Wilcoxon rank sum test with Bonferroni correction, and cell types were manually annotated based on the cluster markers. A marker-cluster heatmap was generated with the R pheatmap(v1.0.12) package, and the marker genes of each cluster were used to performe Gene Ontology (GO) enrichment analysis with clusterProfiler(v3.14.3) R package.To calculate the sample composition based on cell type, the number of cells for each cell type from each sample were counted. The counts were then divided by the total number of cells for each sample and scaled to 100% for each cell type.

The output filtered gene expression matrices of human data were analyzed by the Seurat package (v3.0.2). Cells with <800 transcripts and >7500 transcripts detected were first filtered out as low-quality cells. After removal of low-quality cells, gene expression matrices were normalized by the NormalizeData function and 2000 features with high cell-to-cell variation were calculated using the FindVariableFeatures function. To reduce dimensionality of the datasets, the RunPCA function was conducted with default parameters on linear-transformation scaled data generated by the ScaleData function. Next, the ElbowPlot functions were used to identify the true dimensionality (1:20) of each dataset. Next, we clustered cells using the FindNeighbors and FindClusters functions by setting clustering parameter resolution to 0.4，Finally, the function RunUMAP with dimensions parameters (1:20) in Seurat was used to reduce high-dimension into two-dimension (2D) for visualization.

**Cell-type subclustering**

Subclustering was performed on Neurons-3 of mouse data. The same functions described above were used to obtain the subclusters. After PCA to reduce dimensionality and build k-nearest neighbor graphs (k = 15) of the cells with the function FindNeighbors based on the Euclidean distance in the 50-dimensional PC space, the main cell cluster was identified using the Louvain-Jaccard graph-based method. We first set the clustering parameter resolution to 0.3 for the function FindClusters in Seurat. Last, the function RunUMAP with dimensions parameters (1:15) in Seurat was used to reduce high-dimension into two-dimension (2D) for visualization. We also run the Seurat FindAllMarkers function with the default parameters to identify the genes specifically expressed in each subcluster. To calculate the composition of different groups cells for the subclusters, the numbers of cells in each group for each subcluster were counted.

**DEGs analysis**

Differentially expressed genes (DEGs) testing of two group with each cell type in mouse data were performed using the FindMarkers function in Seurat. The significance of the differences in gene expression was determined using the Wilcoxon rank sum test with Bonferroni correction. The differences genes of two group in each subcluster were determined based on following criteria: 1) expressed in more than 10% of the cells within either or both two groups; 2) |log_2_FC| > 0.25; 3) Wilcoxon rank sum test adjusted p-value < 0.05.

**GO enrichment analysis**

Enrichment scores (p-values) for selected numbers of GO annotations were calculated by clusterProfiler(v3.14.3) R package with a hyper-geometrical statistical test with a threshold of 0.05, and the Benjamini-Hochberg method was used to estimate the false discovery rate (FDR). Enrichment was calculated for the input DEGs in subcluster. The background in mouse data was all the genes listed in the database of org.Mm.eg.db, and the background in human data was all the genes listed in the database of org.Hs.eg.db. Lastly, barplot function was run for visualization.

**Pseudotime trajectory construction**

The trajectory analysis was performed using the Monocle2 R package (v2.14.0) to reveal cell state transitions in mouse Microglia clusters. Briefly, we used raw count matrices for cells in the intended cell types and the negbinomial.size parameter to create a CellDataSet object in the default setting. The data were normalized by the estimateSizeFactors and estimateDispersions functions with the default parameters. We used the differentially expressed genes (DEGs) with qval <1e-2 of Microglia clusters through differentialGeneTest function with the default parameters to sort the cells in pseudo-time order. Dimensional reduction and cell ordering were performed using the DDRTree method and the orderCells function. Lastly, plot_cell_trajectory function was run for visualization. The DEGs changes along the pseudo-time in different branch were also determined with the BEAM function with default parameter, and the DEGs with the adjusted P value(qval <1e-4) were used to plot the heatmap showing the bifurcation expression patterns with plot_genes_branched_heatmap function.

The trajectory analysis was performed using the Monocle2 R package (v2.14.0) to reveal cell state transitions in human Microglia clusters. The raw counts for cells in the intended cell types were extracted and normalized by the estimateSizeFactors and estimateDispersions functions with the default parameters. Only genes matching the thresholds (both of mean expression greater than 0.1 and dispersion_empirical greater than 1.2*dispersion_fit) were used for cell ordering and training the pseudo-time

Trajectory. The orders were determined by the orderCells function and the trajectory was constructed by the reduceDimension function with default parameters. Lastly, plot_cell_trajectory function was run for visualization. The differentially expressed genes(DEGs) changes along the pseudo-time were also determined with the differentialGeneTest function with default parameters, and the DEGs with the adjusted P value(qval <1e-4) were visualized with the function plot_pseudotime_heatmap.

**RNA-velocity analysis**

Analysis of cellular trajectory by RNA velocity was performed using the package scVelo(v0.2.3) using dynamical modeling. To estimate the RNA velocities in Microglia clusters, velocyto was used to distinguish unspliced and spliced messenger RNAs in each sample. The python package scVelo was then used to recover the directed dynamic information by leveraging RNA-splicing information. Specifically, the data were first normalized using the filter_and_normalize function with the following parameter settings: min_shared_counts 30, n_top_genes 1000. The first- and second-order moments were computed for velocity estimation using the moments function with the following parameter settings: n_pcs 30, n_neighbors 30. The velocity vectors were obtained using the velocity function of dynamical modeling. The velocities were projected into a lower-dimensional embedding using the velocity_ graph function, and build k-nearest neighbor graphs of the cells using the neighbors function with the following parameter settings: n_neighbors 10, n_pcs 40. Finally, the velocities were visualized in the UMAP embedding using the umap function with default parameters.

**Cell-cell ligand-receptor** **interaction analysis**

CellphoneDB (v2.1.7) was applied for ligand-receptor analysis. The normalized counts and cell type annotation for each cell were imputed into CellphoneDB to determine the potential ligand-receptor pairs. Interactions pairs with pvalue>0.05 were filtered out from further analysis. First, the interaction of Microglia clusters and Neurons-3 cluster in mouse data were analysed. Next, two runs were performed on two groups of mouse cells between Neurons-3 subclusters in mouse data. Lastly, the interaction of Microglia clusters and Neurons cluster in human data were analysed. Selected specific pairs were plotted by dot_plot function in CellphoneDB with default parameters. In mouse data, we used homologous genes in mouse and humans for this analysis.
